# Supplementary material for: Cost-effectiveness of HPV vaccination in 195 countries: A meta-regression analysis
Source: PLoS One. 2021 Dec 20;16(12):e0260808. doi: 10.1371/journal.pone.0260808 (PMC8687557; doi:10.1371/journal.pone.0260808)
Supplement: S2 Table — (DOCX) [file pone.0260808.s002.docx]

**S2 Table. Selected characteristics of cost-effectiveness articles on human papillomavirus vaccines included in the analysis**

| **Title** | **Year** | **Countries** | **Time horizon** | **DALY/**  **QALY discount rate** | **Costs discount rate** | **Vaccine types** | **Vaccine sexes** | **Vaccine coverage (%)** | **Perspective** | **Number of ratios in Tufts dataset** | **Minimum ICER (2017 US$ per unit change in DALY or QALY)** | **Maximum ICER (2017 US$ per unit change in DALY or QALY)** |
| --- | --- | --- | --- | --- | --- | --- | --- | --- | --- | --- | --- | --- |
| The potential cost-effectiveness of prophylactic human papillomavirus vaccines in Canada[1] | 2007 | Canada | lifetime | 3 | 3 | quadrivalent | female | 100 | healthcare payer | 1 | 18 825 | 18 825 |
| Cost-effectiveness of quadrivalent human papillomavirus (HPV) vaccination in Mexico: a transmission dynamic model-based evaluation[2] | 2007 | Mexico | lifetime | 3 | 3 | quadrivalent | female | 70 | healthcare payer | 1 | 2697 | 2697 |
| Model for assessing human papillomavirus vaccination strategies[3] | 2007 | United States | lifetime | 3 | 3 | quadrivalent | both | 70 | healthcare payer | 5 | 3654 | 14 116 |
| Cost-effectiveness analysis of the introduction of a quadrivalent human papillomavirus vaccine in France[4] | 2008 | France | lifetime | 1 5 | 3 5 | quadrivalent | female | 80 | healthcare payer | 2 | 10 778 | 17 702 |
| Cost-effectiveness analysis of adding a quadrivalent HPV vaccine to the cervical cancer screening programme in Switzerland[5] | 2008 | Switzerland | lifetime | 1 5 | 3 | quadrivalent | female | 80 | healthcare payer | 1 | 26 816 | 26 816 |
| The cost-effectiveness of a quadrivalent human papillomavirus vaccine in Taiwan[6] | 2008 | Taiwan | lifetime | 3 | 3 | quadrivalent | female | 80 | healthcare payer | 2 | 14 427 | 15 374 |
| Assessment of the cost-effectiveness of a quadrivalent HPV vaccine in Norway using a dynamic transmission model[7] | 2008 | Norway | lifetime | 3 5 | 3 5 | quadrivalent | female | 90 | healthcare payer | 2 | 7278 | 9597 |
| Economic evaluation of human papillomavirus vaccination in the United Kingdom[8] | 2008 | United Kingdom | lifetime | 3 5 | 3 5 | quadrivalent | female | 80 | healthcare payer | 1 | 51 249 | 51 249 |
| Adding a quadrivalent human papillomavirus vaccine to the UK cervical cancer screening programme: a cost-effectiveness analysis[9] | 2008 | United Kingdom | lifetime | 3 5 | 3 5 | quadrivalent | female | 85 | healthcare payer | 1 | 33 678 | 33 678 |
| A multi-type HPV transmission model[10] | 2008 | United States | lifetime | 3 | 3 | quadrivalent | both | 70 | healthcare payer | 4 | 3654 | 14 963 |
| Cost-effectiveness of human papillomavirus vaccination in the United States[11] | 2008 | United States | lifetime | 3 | 3 | bivalent, quadrivalent | female | 70 | healthcare payer | 6 | 6579 | 18 153 |
| The epidemiological and economic impact of a quadrivalent human papillomavirus vaccine (6/11/16/18) in the UK[12] | 2008 | United Kingdom | lifetime | 3 5 | 3 5 | quadrivalent | both | 80 | healthcare payer | 4 | 9130 | 9638 |
| Mathematical models of cervical cancer prevention in the Asia Pacific region[13] | 2008 | China, Thailand, Indonesia, Yemen, Afghanistan, Pakistan, Kyrgyzstan, Georgia, Armenia, Uzbekistan, Tajikistan, Azerbaijan, Cambodia, Vietnam, Mongolia, Laos, India, Bangladesh, Bhutan, Nepal, Myanmar, South Korea, Sri Lanka, Timor-Leste, Japan | lifetime | 3 | 3 | bivalent | female | 70 | limited societal | 26 | 36 | 1676 |
| Mathematical models of cervical cancer prevention in Latin America and the Caribbean[14] | 2008 | Jamaica, Dominican Republic, Trinidad And Tobago, Barbados, Cuba, The Bahamas, Belize, Nicaragua, El Salvador, Guatemala, Honduras, Mexico, Panama, Costa Rica, Bolivia, Paraguay, Peru, Guyana, Ecuador, Colombia, Venezuela, Suriname, Chile, Brazil, Argentina, Uruguay, Haiti | lifetime | 3 | 3 | bivalent | female | 70 | limited societal | 27 | cost-saving | 480 |
| Health and economic outcomes of HPV 16,18 vaccination in 72 GAVI-eligible countries[15] | 2008 | The Gambia, Indonesia, Cuba, Nicaragua, Honduras, Bolivia, Guyana, Haiti, Yemen, Afghanistan, Pakistan, Kyrgyzstan, Georgia, Armenia, Uzbekistan, Tajikistan, Azerbaijan, Cambodia, Vietnam, Mongolia, Laos, India, Bangladesh, Bhutan, Nepal, Myanmar, South Korea, Sri Lanka, Timor-Leste, Benin, Burkina Faso, Cameroon, Chad, Comoros, Ghana, Guinea, Guinea-Bissau, Liberia, Madagascar, Mali, Mauritania, Niger, Nigeria, Sao Tome And Principe, Senegal, Sierra Leone, Togo, Burundi, Central African Republic, Dr Congo, Cote D'Ivoire, Eritrea, Ethiopia, Kenya, Lesotho, Malawi, Mozambique, Rwanda, Tanzania, Uganda, Zambia, Zimbabwe, Djibouti, Somalia, Angola, South Sudan, Moldova, Ukraine, Solomon Islands, Papua New Guinea, Kiribati | lifetime | 3 | 3 | bivalent | female | 70 | healthcare payer | 73 | cost-saving | 665 |
| A cost-utility analysis of adding a bivalent or quadrivalent HPV vaccine to the Irish cervical screening programme[16] | 2009 | Ireland | lifetime | 4 | 4 | quadrivalent, bivalent | female | 90 | healthcare payer | 2 | 28 649 | 34 425 |
| Cost-effectiveness of human papilloma virus vaccination in Iceland[17] | 2009 | Iceland | lifetime | 3 | 3 | bivalent | female | 90 | healthcare payer | 1 | 24 471 | 24 471 |
| A cost-utility analysis of cervical cancer vaccination in preadolescent Canadian females[18] | 2009 | Canada | lifetime | 3 | 3 | bivalent | female | 75 | healthcare payer | 4 | 16700 | 28 341 |
| Cost-effectiveness evaluation of a quadrivalent human papillomavirus vaccine in Belgium[19] | 2009 | Belgium | lifetime | 1 5 | 3 | quadrivalent | female | 80 | healthcare payer | 1 | 13 914 | 13 914 |
| Cost-effectiveness analysis of a cervical cancer vaccine in five Latin American countries[20] | 2009 | Chile, Peru, Mexico, Argentina, Brazil | lifetime | 3 | 3 | quadrivalent | female | 100 | healthcare payer | 5 | 5474 | 21 132 |
| Cost-effectiveness analysis of human papillomavirus vaccination in the Netherlands[21] | 2009 | Netherlands | lifetime | 3 | 3 | quadrivalent | female | 85 | societal | 1 | 65 406 | 65 406 |
| HPV16/18 vaccination to prevent cervical cancer in the Netherlands: Model-based cost-effectiveness[22] | 2009 | Netherlands | 88 years | 1 5 | 4 | bivalent | female | 85 | healthcare payer | 9 | 21 812 | 34 793 |
| Cost-effectiveness of human papillomavirus vaccination for prevention of cervical cancer in Taiwan[23] | 2010 | Taiwan | lifetime | 3 | 3 | bivalent | female | 100 | healthcare payer | 1 | 15 509 | 15 509 |
| Human papillomavirus transmission and cost-effectiveness of introducing quadrivalent HPV vaccination in Denmark[24] | 2010 | Denmark | 62 years | 3 | 3 | quadrivalent | both | 70 | healthcare payer | 3 | 2478 | 24 149 |
| Cost-effectiveness analysis of prophylactic cervical cancer vaccination in Japanese women[25] | 2010 | Japan | lifetime | 3 | 3 | bivalent | female | 24 | healthcare payer | 1 | 15 412 | 15 412 |
| Cost-effectiveness analysis of HPV vaccination alongside cervical cancer screening programme in Slovenia[26] | 2010 | Slovenia | lifetime | 5 | 5 | quadrivalent | female | 80 | healthcare payer | 1 | 28 561 | 28 561 |
| Economic evaluation of policy options for prevention and control of cervical cancer in Thailand[27] | 2011 | Thailand | lifetime | 3 | 3 | bivalent | female | 100 | societal | 10 | 5389 | 7144 |
| Until which age should women be vaccinated against hpv infection? Recommendation based on cost-effectiveness analyses[28] | 2011 | Netherlands | lifetime | 1 5 | 4 | bivalent | female | 100 | healthcare payer | 5 | 23 442 | 61 374 |
| Tthe clinical benefit and cost-effectiveness of human papillomavirus vaccination for adult women in the Netherlands[29] | 2011 | Netherlands | lifetime | 1 5 | 4 | bivalent | female | 50 | healthcare payer | 1 | 26 536 | 26 536 |
| Comparative evaluation of the potential impact of Rotavirus versus HPV vaccination in GAVI-eligible countries: A preliminary analysis focused on the relative disease burden[30] | 2011 | The Gambia, Indonesia, Cuba, Nicaragua, Honduras, Bolivia, Guyana, Haiti, Yemen, Afghanistan, Pakistan, Kyrgyzstan, Georgia, Armenia, Uzbekistan, Tajikistan, Azerbaijan, Cambodia, Vietnam, Mongolia, Laos, India, Bangladesh, Bhutan, Nepal, Myanmar, South Korea, Sri Lanka, Timor-Leste, Benin, Burkina Faso, Cameroon, Chad, Comoros, Ghana, Guinea, Guinea-Bissau, Liberia, Madagascar, Mali, Mauritania, Niger, Nigeria, Sao Tome And Principe, Senegal, Sierra Leone, Togo, Burundi, Central African Republic, Dr Congo, Congo, Cote D'Ivoire, Eritrea, Ethiopia, Kenya, Lesotho, Malawi, Mozambique, Rwanda, Tanzania, Uganda, Zambia, Zimbabwe, Djibouti, Somalia, Sudan, Angola, Moldova, Ukraine, Solomon Islands, Papua New Guinea, Kiribati | lifetime | 3 | 3 | bivalent | both | 70 | healthcare payer | 72 | cost-saving | 727 |
| Cost-effectiveness of different human papillomavirus vaccines in Singapore[31] | 2011 | Singapore | lifetime | 3 | 3 | quadrivalent, bivalent | female | 100 | healthcare payer | 2 | 7163 | 8060 |
| Comparing bivalent and quadrivalent human papillomavirus vaccines: Economic evaluation based on transmission model[32] | 2011 | United Kingdom | lifetime | 3 5 | 3 5 | bivalent, quadrivalent | both | 80 | healthcare payer | 2 | 24 643 | 41 314 |
| Cost-effectiveness of a cervical screening program with human papillomavirus vaccine[33] | 2011 | New Zealand | lifetime | 3 | 3 | quadrivalent | female | 30, 50, 70, 90 | healthcare payer | 8 | 2892 | 8256 |
| Novel health economic evaluation of a vaccination strategy to prevent HPV-related diseases: The BEST study[34] | 2012 | Italy | lifetime | 1 5 | 3 | quadrivalent | female | 85 | healthcare payer | 1 | 14 438 | 14 438 |
| Cost and effectiveness evaluation of prophylactic HPV vaccine in developing countries[35] | 2012 | Thailand | lifetime | 3 | 3 | quadrivalent | female | 100 | healthcare payer | 1 | 5279 | 5279 |
| Estimated health and economic impact of quadrivalent HPV (types 6/11/16/18) vaccination in Brazil using a transmission dynamic model[36] | 2012 | Brazil | lifetime | 3 | 3 | quadrivalent | female | 85 | healthcare payer | 2 | 240 | 457 |
| The cost efficiency of HPV vaccines is significantly underestimated due to omission of conisation-associated prematurity with neonatal mortality and morbidity[37] | 2012 | Germany | 30 years | 3 | 3 | bivalent | female | 50, 60 | healthcare payer | 2 | 54 608 | 73 306 |
| Cost-effectiveness of adding vaccination with the as04-adjuvanted human papillomavirus 16/18 vaccine to cervical cancer screening in Hungary[38] | 2012 | Hungary | 88 years | 3 7 | 3 7 | bivalent | female | 80 | healthcare payer | 1 | 30 912 | 30 912 |
| Time for change? An economic evaluation of integrated cervical screening and HPV immunization programs in Canada[39] | 2012 | Canada | 80 years | 3 | 3 | bivalent | both | 80 | healthcare payer | 2 | 5244 | 5447 |
| Economic modelling assessment of the hpv quadrivalent vaccine in Brazil: A dynamic individual-based approach[40] | 2012 | Brazil | lifetime | 5 | 5 | quadrivalent | female | 70 | healthcare payer | 1 | cost-saving | cost-saving |
| Cost-effectiveness of vaccination with a quadrivalent HPV vaccine in Germany using a dynamic transmission model[41] | 2012 | Germany | lifetime | 3 | 3 | quadrivalent | female | 55 | healthcare payer | 1 | 6873 | 6873 |
| Cost-effectiveness of the prophylactic HPV vaccine: An application to the Netherlands taking non-cervical cancers and cross-protection into account[42] | 2013 | Netherlands | lifetime | 1 5 | 4 | bivalent | female | 100 | healthcare payer | 2 | 6850 | 8413 |
| Model-based impact and cost-effectiveness of cervical cancer prevention in Sub-Saharan Africa[43] | 2013 | The Gambia, Benin, Burkina Faso, Cameroon, Cape Verde, Chad, Comoros, Equatorial Guinea, Gabon, Ghana, Guinea, Guinea-Bissau, Liberia, Madagascar, Mali, Mauritania, Mauritius, Niger, Nigeria, Sao Tome And Principe, Senegal, Seychelles, Sierra Leone, Togo, Botswana, Burundi, Central African Republic, Dr Congo, Congo, Cote D'Ivoire, Eritrea, Ethiopia, Kenya, Lesotho, Malawi, Mozambique, Namibia, Rwanda, South Africa, Swaziland, Tanzania, Uganda, Zambia, Zimbabwe, Djibouti, Somalia, Sudan, Angola | lifetime | 3 | 3 | bivalent | female | 70 | limited societal | 48 | cost-saving | 369 |
| Comparative cost-effectiveness of the quadrivalent and bivalent human papillomavirus vaccines: A transmission-dynamic modeling study[44] | 2013 | Canada | 70 years | 3 | 3 | quadrivalent, bivalent | female | 80 | healthcare payer | 2 | 12 858 | 16 712 |
| Model-based impact and cost-effectiveness of cervical cancer prevention in the extended Middle East and North Africa (EMENA)[45] | 2013 | Yemen, Afghanistan, Pakistan, Algeria, Bahrain, Egypt, Iran, Iraq, Jordan, Kuwait, Lebanon, Libya, Morocco, Oman, Qatar, Saudi Arabia, Syria, Tunisia, Turkey, United Arab Emirates | lifetime | 3 | 3 | bivalent | female | 70 | limited societal | 20 | cost-saving | 1726 |
| The epidemiological and economic impact of a quadrivalent human papillomavirus (HPV) vaccine in Estonia[46] | 2013 | Estonia | lifetime | 3 | 3 | quadrivalent | female | 85 | healthcare payer | 1 | 6328 | 6328 |
| Cost-effectiveness analysis of the bivalent and quadrivalent human papillomavirus vaccines from a societal perspective in Colombia[47] | 2013 | Colombia | lifetime | 3 | 3 | quadrivalent, bivalent | female | 80 | limited societal | 2 | 27 162 | 32 231 |
| Inclusion of the benefits of enhanced cross-protection against cervical cancer and prevention of genital warts in the cost-effectiveness analysis of human papillomavirus vaccination in the Netherlands[48] | 2013 | Netherlands | lifetime | 1 5 | 4 | bivalent, quadrivalent | female | 50 | healthcare payer | 2 | 37 417 | 43 418 |
| Universal vaccination with the quadrivalent HPV vaccine in Austria: Impact on virus circulation, public health and cost-effectiveness analysis[49] | 2014 | Austria | lifetime | 3 | 3 | quadrivalent | both | 65 | healthcare payer | 3 | 12 669 | 33 717 |
| Health economic analysis of human papillomavirus vaccines in women of Chile: perspective of the health care payer using a markov model[50] | 2014 | Chile | lifetime | 6 | 6 | bivalent, quadrivalent | female | 95 | healthcare payer | 2 | 129 | 606 |
| Cost-effectiveness evaluation of quadrivalent human papilloma virus vaccine for HPV-related disease in Iran[51] | 2014 | Iran | lifetime | 3 | 3 | quadrivalent | female | 70 | healthcare payer | 1 | 22 353 | 22 353 |
| Cost-effectiveness of female human papillomavirus vaccination in 179 countries: A PRIME modelling study[52] | 2014 | Iran, Benin, Botswana, Burkina Faso, Burundi, Cameroon, Cape Verde, Central African Republic, Chad, Comoros, Cote D'Ivoire, Dr Congo, Djibouti, Equatorial Guinea, Eritrea, Ethiopia, Gabon, The Gambia, Ghana, Guinea, Guinea-Bissau, Kenya, Lesotho, Liberia, Madagascar, Malawi, Mauritania, Mozambique, Namibia, Niger, Nigeria, Rwanda, Senegal, Sierra Leone, South Africa, Sudan, Swaziland, Tanzania, Togo, Uganda, Zambia, Zimbabwe, Mali, Angola, Brazil, Albania, Argentina, India, Israel, Ecuador, Congo, Somalia, China, Afghanistan, Algeria, Armenia, Australia, Austria, Azerbaijan, The Bahamas, Bahrain, Bangladesh, Barbados, Belarus, Belgium, Belize, Bhutan, Bolivia, Bosnia And Herzegovina, Brunei, Bulgaria, Cambodia, Canada, Chile, Colombia, Costa Rica, Croatia, Cuba, Cyprus, Czech Republic, Denmark, Dominican Republic, Egypt, El Salvador, Estonia, Fiji, Finland, France, Georgia, Germany, Greece, Grenada, Guatemala, Guyana, Haiti, Honduras, Hungary, Iceland, Indonesia, Iraq, Ireland, Italy, Jamaica, Japan, Jordan, Kazakhstan, South Korea, Kuwait, Kyrgyzstan, Laos, Latvia, Lebanon, Libya, Lithuania, Luxembourg, Macedonia, Malaysia, Maldives, Malta, Mauritius, Mexico, Federated States Of Micronesia, Moldova, Mongolia, Montenegro, Morocco, Myanmar, Nepal, Netherlands, New Zealand, Nicaragua, Norway, Oman, Pakistan, Panama, Papua New Guinea, Paraguay, Peru, Philippines, Poland, Portugal, Qatar, Romania, Russia, Samoa, Sao Tome And Principe, Saudi Arabia, Serbia, Singapore, Slovakia, Slovenia, Solomon Islands, Spain, Sri Lanka, Saint Lucia, Saint Vincent And The Grenadines, Suriname, Sweden, Switzerland, Syria, Tajikistan, Thailand, Timor-Leste, Tonga, Trinidad And Tobago, Tunisia, Turkey, Turkmenistan, Ukraine, United Arab Emirates, United Kingdom, United States, Uruguay, Uzbekistan, Vanuatu, Venezuela, Vietnam, Yemen | lifetime | 3 | 3 | bivalent | female | 100 | healthcare payer | 186 | 100 | 859 624 |
| Potential cost-effectiveness of the nonavalent human papillomavirus (HPV) vaccine[53] | 2014 | Canada | 70 years | 3 | 3 | quadrivalent | female | 80 | healthcare payer | 1 | 12 858 | 12 858 |
| Prevention of hpv-related cancers in Norway: cost-effectiveness of expanding the HPV vaccination program to include pre-adolescent boys[54] | 2014 | Norway | lifetime | 4 | 4 | quadrivalent | both | 71 | limited societal | 2 | 5602 | 67 343 |
| Cost-effectiveness and equity impacts of three HPV vaccination programmes for school-aged girls in New Zealand[55] | 2014 | New Zealand | lifetime | 3 | 3 | quadrivalent | female | 73, 93, 56 | healthcare payer | 3 | 2376 | 17 328 |
| Is expanding HPV vaccination programs to include school-aged boys likely to be value-for-money: A cost-utility analysis in a country with an existing school-girl program[56] | 2014 | New Zealand | lifetime | 3 | 3 | quadrivalent | both | 73, 56.0 | healthcare payer | 4 | 14 414 | 41 864 |
| Comparing the cost-effectiveness of two- and three-dose schedules of human papillomavirus vaccination: A transmission-dynamic modelling study[57] | 2014 | Canada | 70 years | 3 | 3 | quadrivalent | both | 80 | healthcare payer | 4 | 8614 | 16 309 |
| Cost-effectiveness of HPV vaccination in Belize[58] | 2015 | Belize | lifetime | 3 | 3 | quadrivalent | female | 95 | limited societal | 1 | 462 | 462 |
| Cost-effectiveness analysis of the introduction of the human papillomavirus vaccine in Honduras[59] | 2015 | Honduras | lifetime | 3 | 3 | bivalent | female | 95 | limited societal | 1 | 894 | 894 |
| Cost-effectiveness of a bivalent human papillomavirus vaccination program in Japan[60] | 2015 | Japan | lifetime | 5 | 5 | bivalent | female | 80 | healthcare payer | 1 | 21 171 | 21 171 |
| Present and future of cervical cancer prevention in Spain: A cost-effectiveness analysis[61] | 2015 | Spain | 74 years | 3 | 3 | bivalent | female | 70 | limited societal | 1 | cost-saving | cost-saving |
| Too late to vaccinate? The incremental benefits and cost-effectiveness of a delayed catch-up program using the 4-valent human papillomavirus vaccine in Norway[62] | 2015 | Norway | lifetime | 4 | 4 | quadrivalent | female | 50 | limited societal | 4 | 34 175 | 217 380 |
| Cost-effectiveness analysis of human papillomavirus vaccination in South Africa accounting for human immunodeficiency virus prevalence[63] | 2015 | South Africa | lifetime | 5 | 5 | bivalent | female | 100 | healthcare payer | 1 | 7325 | 7325 |
| The value of male human papillomavirus vaccination in preventing cervical cancer and genital warts in a low-resource setting[64] | 2015 | Vietnam | lifetime | 3 | 3 | quadrivalent | both | 90 | limited societal | 1 | 2358 | 2358 |
| Cost-effectiveness analysis of introducing universal human papillomavirus vaccination of girls aged 11 years into the national immunization program in Brazil[65] | 2015 | Brazil | lifetime | 5 | 5 | bivalent | female | 50 | limited societal | 1 | 8340 | 8340 |
| Cost-effectiveness of human papillomavirus vaccination in Germany[66] | 2016 | Germany | lifetime | 3 | 3 | bivalent, quadrivalent | female | 50 | limited societal | 2 | 13 365 | 41 725 |
| Cost-effectiveness analysis of different types of human papillomavirus vaccination combined with a cervical cancer screening program in mainland China[67] | 2016 | China | lifetime | 3 | 3 | quadrivalent, bivalent | female | 20 | limited societal | 2 | 5585 | 80 507 |
| Health economic evaluation of human papillomavirus vaccines in women from Venezuela by a lifetime markov cohort model[68] | 2016 | Venezuela | lifetime | 5 | 5 | quadrivalent, bivalent | female | 95 | healthcare payer | 2 | cost-saving | cost-saving |
| Modelling the effects of quadrivalent human papillomavirus (hpv) vaccination in Puerto Rico[69] | 2016 | United States | lifetime | 3 | 3 | quadrivalent | both | 23 5, 45, 70 | healthcare payer | 3 | 2062 | 4772 |
| Cost-effectiveness of HPV vaccination in the context of high cervical cancer incidence and low screening coverage[70] | 2016 | Estonia | lifetime | 5 | 5 | bivalent, quadrivalent | female | 70 | healthcare payer | 2 | 15 832 | 15 900 |
| The economic evaluation of human papillomavirus vaccination strategies against cervical cancer in women in Lao PDR: A mathematical modelling approach[71] | 2016 | Laos | lifetime | 3 | 3 | bivalent | both | 70 | healthcare payer | 4 | 737 | 2074 |
| Health and economic impact of switching from a 4-valent to a 9-valent HPV vaccination program in the United States[72] | 2016 | United States | 70 years | 3 | 3 | quadrivalent | both | 50 | healthcare payer | 1 | 8179 | 8179 |
| Cost-effectiveness of two-dose human papillomavirus vaccination in Singapore[73] | 2016 | Singapore | lifetime | 3 | 3 | bivalent | female | 90 | healthcare payer | 1 | 9518 | 9518 |
| Cost-effectiveness of HPV vaccination regime: Comparing twice versus thrice vaccinations dose regime among adolescent girls in Malaysia[74] | 2016 | Malaysia | lifetime | 3 | 3 | bivalent | female | 100 | healthcare payer | 1 | cost-saving | cost-saving |
| Cervical cancer treatment costs and cost-effectiveness analysis of human papillomavirus vaccination in Vietnam: A PRIME modeling study[75] | 2017 | Vietnam | lifetime | 3 | 3 | quadrivalent, bivalent | female | 95 | healthcare payer | 21 | 18 | 8649 |
| Cost-effectiveness of human papilloma virus (HPV) vaccination in nigeria: A decision analysis using pragmatic parameter estimates for cost and programme coverage[76] | 2017 | Nigeria | lifetime | 3 | 3 | bivalent | female | 66 | healthcare payer | 9 | 8175 | 17 764 |

**S2 Table References**

1. Brisson M, Van de Velde N, De Wals P, Boily M-C. The potential cost-effectiveness of prophylactic human papillomavirus vaccines in Canada. Vaccine 2007; 25: 5399–408.

2. Insinga RP, Dasbach EJ, Elbasha EH, Puig A, Reynales-Shigematsu LM. Cost-effectiveness of quadrivalent human papillomavirus (HPV) vaccination in Mexico: a transmission dynamic model-based evaluation. Vaccine 2007; 26: 128–39.

3. Elbasha EH, Dasbach EJ, Insinga RP. Model for assessing human papillomavirus vaccination strategies. Emerging Infect Dis 2007; 13: 28–41.

4. Bergeron C, Largeron N, McAllister R, Mathevet P, Remy V. Cost-effectiveness analysis of the introduction of a quadrivalent human papillomavirus vaccine in France. Int J Technol Assess Health Care 2008; 24: 10–9.

5. Szucs TD, Largeron N, Dedes KJ, Rafia R, Bénard S. Cost-effectiveness analysis of adding a quadrivalent HPV vaccine to the cervical cancer screening programme in Switzerland. Curr Med Res Opin 2008; 24: 1473–83.

6. Dasbach EJ, Insinga RP, Yang YC, Pwu R-F, Lac C, Elbasha EH. The cost-effectiveness of a quadrivalent human papillomavirus vaccine in Taiwan. Asian Pac J Cancer Prev 2008; 9: 459–66.

7. Dasbach EJ, Largeron N, Elbasha EH. Assessment of the cost-effectiveness of a quadrivalent HPV vaccine in Norway using a dynamic transmission model. Expert Rev Pharmacoecon Outcomes Res 2008; 8: 491–500.

8. Jit M, Choi YH, Edmunds WJ. Economic evaluation of human papillomavirus vaccination in the United Kingdom. BMJ 2008; 337: a769.

9. Kulasingam SL, Benard S, Barnabas RV, Largeron N, Myers ER. Adding a quadrivalent human papillomavirus vaccine to the UK cervical cancer screening programme: A cost-effectiveness analysis. Cost Eff Resour Alloc 2008; 6: 4.

10. Elbasha EH, Dasbach EJ, Insinga RP. A multi-type HPV transmission model. Bull Math Biol 2008; 70: 2126–76.

11. Chesson HW, Ekwueme DU, Saraiya M, Markowitz LE. Cost-effectiveness of human papillomavirus vaccination in the United States. Emerging Infect Dis 2008; 14: 244–51.

12. Dasbach EJ, Insinga RP, Elbasha EH. The epidemiological and economic impact of a quadrivalent human papillomavirus vaccine (6/11/16/18) in the UK. BJOG 2008; 115: 947–56.

13. Goldie SJ, Diaz M, Kim S-Y, Levin CE, Van Minh H, Kim JJ. Mathematical models of cervical cancer prevention in the Asia Pacific region. Vaccine 2008; 26 Suppl 12: M17-29.

14. Goldie SJ, Diaz M, Constenla D, Alvis N, Andrus JK, Kim S-Y. Mathematical models of cervical cancer prevention in Latin America and the Caribbean. Vaccine 2008; 26 Suppl 11: L59-72.

15. Goldie SJ, O’Shea M, Campos NG, Diaz M, Sweet S, Kim S-Y. Health and economic outcomes of HPV 16,18 vaccination in 72 GAVI-eligible countries. Vaccine 2008; 26: 4080–93.

16. Dee A, Howell F. A cost-utility analysis of adding a bivalent or quadrivalent HPV vaccine to the Irish cervical screening programme. Eur J Public Health 2010; 20: 213–9.

17. Oddsson K, Johannsson J, Asgeirsdottir TL, Gudnason T. Cost-effectiveness of human papilloma virus vaccination in Iceland. Acta Obstet Gynecol Scand 2009; 88: 1411–6.

18. Anonychuk AM, Bauch CT, Merid MF, Van Kriekinge G, Demarteau N. A cost-utility analysis of cervical cancer vaccination in preadolescent Canadian females. BMC Public Health 2009; 9: 401.

19. Annemans L, Rémy V, Oyee J, Largeron N. Cost-effectiveness evaluation of a quadrivalent human papillomavirus vaccine in Belgium. Pharmacoeconomics 2009; 27: 231–45.

20. Colantonio L, Gómez JA, Demarteau N, Standaert B, Pichón-Rivière A, Augustovski F. Cost-effectiveness analysis of a cervical cancer vaccine in five Latin American countries. Vaccine 2009; 27: 5519–29.

21. de Kok IMCM, van Ballegooijen M, Habbema JDF. Cost-effectiveness analysis of human papillomavirus vaccination in the Netherlands. J Natl Cancer Inst 2009; 101: 1083–92.

22. Coupé VMH, van Ginkel J, de Melker HE, Snijders PJF, Meijer CJLM, Berkhof J. HPV16/18 vaccination to prevent cervical cancer in The Netherlands: model-based cost-effectiveness. Int J Cancer 2009; 124: 970–8.

23. Liu P-H, Hu F-C, Lee P-I, Chow S-N, Huang C-W, Wang J-D. Cost-effectiveness of human papillomavirus vaccination for prevention of cervical cancer in Taiwan. BMC Health Serv Res 2010; 10: 11.

24. Olsen J, Jepsen MR. Human papillomavirus transmission and cost-effectiveness of introducing quadrivalent HPV vaccination in Denmark. Int J Technol Assess Health Care 2010; 26: 183–91.

25. Konno R, Sasagawa T, Fukuda T, Van Kriekinge G, Demarteau N. Cost-effectiveness analysis of prophylactic cervical cancer vaccination in Japanese women. Int J Gynecol Cancer 2010; 20: 385–92.

26. Obradovic M, Mrhar A, Kos M. Cost-effectiveness analysis of HPV vaccination alongside cervical cancer screening programme in Slovenia. Eur J Public Health 2010; 20: 415–21.

27. Praditsitthikorn N, Teerawattananon Y, Tantivess S, et al. Economic evaluation of policy options for prevention and control of cervical cancer in Thailand. Pharmacoeconomics 2011; 29: 781–806.

28. Westra TA, Rozenbaum MH, Rogoza RM, et al. Until which age should women be vaccinated against HPV infection? Recommendation based on cost-effectiveness analyses. J Infect Dis 2011; 204: 377–84.

29. Bogaards JA, Coupé VMH, Meijer CJLM, Berkhof J. The clinical benefit and cost-effectiveness of human papillomavirus vaccination for adult women in the Netherlands. Vaccine 2011; 29: 8929–36.

30. Kim S-Y, Sweet S, Chang J, Goldie SJ. Comparative evaluation of the potential impact of rotavirus versus HPV vaccination in GAVI-eligible countries: a preliminary analysis focused on the relative disease burden. BMC Infect Dis 2011; 11: 174.

31. Lee VJ, Tay SK, Teoh YL, Tok MY. Cost-effectiveness of different human papillomavirus vaccines in Singapore. BMC Public Health 2011; 11: 203.

32. Jit M, Chapman R, Hughes O, Choi YH. Comparing bivalent and quadrivalent human papillomavirus vaccines: economic evaluation based on transmission model. BMJ 2011; 343: d5775.

33. Sopina E, Ashton T. Cost-effectiveness of a cervical screening program with human papillomavirus vaccine. Int J Technol Assess Health Care 2011; 27: 290–7.

34. Favato G, Baio G, Capone A, et al. Novel health economic evaluation of a vaccination strategy to prevent HPV-related diseases: the BEST study. Med Care 2012; 50: 1076–85.

35. Termrungruanglert W, Havanond P, Khemapech N, et al. Cost and effectiveness evaluation of prophylactic HPV vaccine in developing countries. Value Health 2012; 15: S29-34.

36. Kawai K, de Araujo GTB, Fonseca M, Pillsbury M, Singhal PK. Estimated health and economic impact of quadrivalent HPV (types 6/11/16/18) vaccination in Brazil using a transmission dynamic model. BMC Infect Dis 2012; 12: 250.

37. Soergel P, Makowski L, Schippert C, Staboulidou I, Hille U, Hillemanns P. The cost efficiency of HPV vaccines is significantly underestimated due to omission of conisation-associated prematurity with neonatal mortality and morbidity. Hum Vaccin Immunother 2012; 8: 243–51.

38. Vokó Z, Nagyjánosi L, Kaló Z. Cost-effectiveness of adding vaccination with the AS04-adjuvanted human papillomavirus 16/18 vaccine to cervical cancer screening in Hungary. BMC Public Health 2012; 12: 924.

39. Tully SP, Anonychuk AM, Sanchez DM, Galvani AP, Bauch CT. Time for change? An economic evaluation of integrated cervical screening and HPV immunization programs in Canada. Vaccine 2012; 30: 425–35.

40. Vanni T, Luz PM, Foss A, Mesa-Frias M, Legood R. Economic modelling assessment of the HPV quadrivalent vaccine in Brazil: a dynamic individual-based approach. Vaccine 2012; 30: 4866–71.

41. Schobert D, Remy V, Schoeffski O. Cost-effectiveness of vaccination with a quadrivalent HPV vaccine in Germany using a dynamic transmission model. Health Econ Rev 2012; 2: 19.

42. Luttjeboer J, Westra TA, Wilschut JC, Nijman HW, Daemen T, Postma MJ. Cost-effectiveness of the prophylactic HPV vaccine: an application to the Netherlands taking non-cervical cancers and cross-protection into account. Vaccine 2013; 31: 3922–7.

43. Kim JJ, Campos NG, O’Shea M, Diaz M, Mutyaba I. Model-based impact and cost-effectiveness of cervical cancer prevention in sub-Saharan Africa. Vaccine 2013; 31 Suppl 5: F60-72.

44. Brisson M, Laprise J-F, Drolet M, et al. Comparative cost-effectiveness of the quadrivalent and bivalent human papillomavirus vaccines: a transmission-dynamic modeling study. Vaccine 2013; 31: 3863–71.

45. Kim JJ, Sharma M, O’Shea M, et al. Model-based impact and cost-effectiveness of cervical cancer prevention in the Extended Middle East and North Africa (EMENA). Vaccine 2013; 31 Suppl 6: G65-77.

46. Uusküla A, Müürsepp A, Kawai K, Raag M, Jürisson M, Pillsbury M. The epidemiological and economic impact of a quadrivalent human papillomavirus (hpv) vaccine in Estonia. BMC Infect Dis 2013; 13: 304.

47. Aponte-González J, Fajardo-Bernal L, Diaz J, Eslava-Schmalbach J, Gamboa O, Hay JW. Cost-effectiveness analysis of the bivalent and quadrivalent human papillomavirus vaccines from a societal perspective in Colombia. PLoS ONE 2013; 8: e80639.

48. Westra TA, Stirbu-Wagner I, Dorsman S, et al. Inclusion of the benefits of enhanced cross-protection against cervical cancer and prevention of genital warts in the cost-effectiveness analysis of human papillomavirus vaccination in the Netherlands. BMC Infect Dis 2013; 13: 75.

49. Bresse X, Goergen C, Prager B, Joura E. Universal vaccination with the quadrivalent HPV vaccine in Austria: impact on virus circulation, public health and cost-effectiveness analysis. Expert Rev Pharmacoecon Outcomes Res 2014; 14: 269–81.

50. Gomez JA, Lepetic A, Demarteau N. Health economic analysis of human papillomavirus vaccines in women of Chile: perspective of the health care payer using a Markov model. BMC Public Health 2014; 14: 1222.

51. Khatibi M, Rasekh HR, Shahverdi Z, Jamshidi HR. Cost-Effectiveness Evaluation of Quadrivalent Human Papilloma Virus Vaccine for HPV-Related Disease in Iran. Iran J Pharm Res 2014; 13: 225–34.

52. Jit M, Brisson M, Portnoy A, Hutubessy R. Cost-effectiveness of female human papillomavirus vaccination in 179 countries: a PRIME modelling study. Lancet Glob Health 2014; 2: e406-414.

53. Drolet M, Laprise J-F, Boily M-C, Franco EL, Brisson M. Potential cost-effectiveness of the nonavalent human papillomavirus (HPV) vaccine. Int J Cancer 2014; 134: 2264–8.

54. Burger EA, Sy S, Nygård M, Kristiansen IS, Kim JJ. Prevention of HPV-related cancers in Norway: cost-effectiveness of expanding the HPV vaccination program to include pre-adolescent boys. PLoS ONE 2014; 9: e89974.

55. Blakely T, Kvizhinadze G, Karvonen T, Pearson AL, Smith M, Wilson N. Cost-effectiveness and equity impacts of three HPV vaccination programmes for school-aged girls in New Zealand. Vaccine 2014; 32: 2645–56.

56. Pearson AL, Kvizhinadze G, Wilson N, Smith M, Canfell K, Blakely T. Is expanding HPV vaccination programs to include school-aged boys likely to be value-for-money: a cost-utility analysis in a country with an existing school-girl program. BMC Infect Dis 2014; 14: 351.

57. Laprise J-F, Drolet M, Boily M-C, et al. Comparing the cost-effectiveness of two- and three-dose schedules of human papillomavirus vaccination: a transmission-dynamic modelling study. Vaccine 2014; 32: 5845–53.

58. Walwyn L, Janusz CB, Clark AD, Prieto E, Waight E, Largaespada N. Cost-effectiveness of HPV vaccination in Belize. Vaccine 2015; 33 Suppl 1: A174-181.

59. Aguilar IBM, Mendoza LO, García O, et al. Cost-effectiveness analysis of the introduction of the human papillomavirus vaccine in Honduras. Vaccine 2015; 33 Suppl 1: A167-173.

60. Connelly LB, Le HND. Cost-effectiveness of a bivalent human papillomavirus vaccination program in Japan. Sex Health 2015; 12: 520–31.

61. Georgalis L, de Sanjosé S, Esnaola M, Bosch FX, Diaz M. Present and future of cervical cancer prevention in Spain: a cost-effectiveness analysis. Eur J Cancer Prev 2016; 25: 430–9.

62. Burger EA, Sy S, Nygård M, Kristiansen IS, Kim JJ. Too late to vaccinate? The incremental benefits and cost-effectiveness of a delayed catch-up program using the 4-valent human papillomavirus vaccine in Norway. J Infect Dis 2015; 211: 206–15.

63. Li X, Stander MP, Van Kriekinge G, Demarteau N. Cost-effectiveness analysis of human papillomavirus vaccination in South Africa accounting for human immunodeficiency virus prevalence. BMC Infect Dis 2015; 15: 566.

64. Sharma M, Sy S, Kim JJ. The value of male human papillomavirus vaccination in preventing cervical cancer and genital warts in a low-resource setting. BJOG 2016; 123: 917–26.

65. Novaes HMD, de Soárez PC, Silva GA, et al. Cost-effectiveness analysis of introducing universal human papillomavirus vaccination of girls aged 11 years into the National Immunization Program in Brazil. Vaccine 2015; 33 Suppl 1: A135-142.

66. Damm O, Horn J, Mikolajczyk RT, et al. Cost-effectiveness of human papillomavirus vaccination in Germany. Cost Eff Resour Alloc 2017; 15: 18.

67. Mo X, Gai Tobe R, Wang L, et al. Cost-effectiveness analysis of different types of human papillomavirus vaccination combined with a cervical cancer screening program in mainland China. BMC Infect Dis 2017; 17: 502.

68. Bardach AE, Garay OU, Calderón M, et al. Health economic evaluation of Human Papillomavirus vaccines in women from Venezuela by a lifetime Markov cohort model. BMC Public Health 2017; 17: 152.

69. Ortiz AP, Ortiz-Ortiz KJ, Ríos M, et al. Modelling the effects of quadrivalent Human Papillomavirus (HPV) vaccination in Puerto Rico. PLoS ONE 2017; 12: e0184540.

70. Võrno T, Lutsar K, Uusküla A, et al. Cost-effectiveness of HPV vaccination in the context of high cervical cancer incidence and low screening coverage. Vaccine 2017; 35: 6329–35.

71. Chanthavilay P, Reinharz D, Mayxay M, et al. The economic evaluation of human papillomavirus vaccination strategies against cervical cancer in women in Lao PDR: a mathematical modelling approach. BMC Health Serv Res 2016; 16: 418.

72. Brisson M, Laprise J-F, Chesson HW, et al. Health and Economic Impact of Switching from a 4-Valent to a 9-Valent HPV Vaccination Program in the United States. J Natl Cancer Inst 2016; 108. DOI:10.1093/jnci/djv282.

73. Tay SK, Lee B-W, Sohn WY, et al. Cost-effectiveness of two-dose human papillomavirus vaccination in Singapore. Singapore Med J 2018; 59: 370–82.

74. Aljunid S, Maimaiti N, Nur AM, Noor MRM, Wan Puteh SE. Cost-effectiveness of HPV vaccination regime: comparing twice versus thrice vaccinations dose regime among adolescent girls in Malaysia. BMC Public Health 2016; 16: 71.

75. Van Minh H, My NTT, Jit M. Cervical cancer treatment costs and cost-effectiveness analysis of human papillomavirus vaccination in Vietnam: a PRIME modeling study. BMC Health Serv Res 2017; 17: 353.

76. Ekwunife OI, Lhachimi SK. Cost-effectiveness of Human Papilloma Virus (HPV) vaccination in Nigeria: a decision analysis using pragmatic parameter estimates for cost and programme coverage. BMC Health Serv Res 2017; 17: 815.
