# Supplementary material for: Cost-effectiveness of HPV vaccination in 195 countries: A meta-regression analysis
Source: PLoS One. 2021 Dec 20;16(12):e0260808. doi: 10.1371/journal.pone.0260808 (PMC8687557; doi:10.1371/journal.pone.0260808)
Supplement: S1 Appendix — (DOCX) [file pone.0260808.s003.docx]

**S1 Appendix. Intervention taxonomy**

##

**Section S1.1: Intervention taxonomy overview**

The analysis required a standardized description of each intervention. The Tufts University’s Cost-Effectiveness Analysis (CEA) Registry and Global CEA registry report an “intervention phrase” that is an unstandardized text variable. For example, in our pilot work with the registries, we found the following three interventions:

• Newer antidepressants: selective serotonin reuptake inhibitors (SSRIs, e.g. fluoxetine)

• Selective serotonin reuptake inhibitors as first-line treatment

• Episodic drug treatment for major depression

When we read the published articles, we learned that these interventions were all SSRIs. From a research perspective, we wanted to create a variable that reports them as the same intervention rather than three different interventions.

We reviewed existing intervention taxonomies to adapt for this analysis, including the World Health Organization International Classification of Health Interventions (ICHI), the ITAX taxonomy focused on capturing intervention features such as adaptability and mechanism of action,[1] and Cochrane’s EPOC taxonomy.[2] After reviewing these existing taxonomies, we thought that they would not allow us to classify health interventions with sufficient detail to distinguish between drivers of ICERs. Therefore, we leveraged the IHME Global Health Data Exchange (GHDx) platform to develop an intervention taxonomy to standardize and categorize each intervention represented in the Tufts registries.

We used Open Refine to group similar interventions together using its natural language processing and text filtering tools.[3] We created separate taxonomies for intervention keywords and intervention details. Intervention keywords represented unique intervention components, whereas intervention details represented intervention attributes (e.g. vaccine type, drug dosage, duration of intervention). For each term in the taxonomy, we added synonyms from each intervention description that were unique to that term to the taxonomy. These synonyms were pulled from the Tufts “Intervention Phrase” or “Intervention Paragraph” variables. When these variables were either too broad to add as synonyms or too vague to determine which keyword to assign them, we returned to the articles to extract additional details on the interventions and updated the intervention descriptions accordingly. We ran a SQL query to export all of the taxonomy terms and synonyms, and created a Python dictionary where the taxonomy terms were keys and the synonyms were values. We used this dictionary to map all of the ratios in the Tufts registries to one or more intervention keywords and intervention details.

**Section S1.2. Guiding principles for intervention taxonomy**

When building out the intervention taxonomy, we developed several guiding principles, including the following:

1. For pharmaceutical interventions, we chose to use the drug class as the most granular level of detail.
2. We excluded information about the specific target population (e.g. age, sex, risk group) and delivery platform from the intervention keywords, as we had processes for capturing these details in additional variables.
3. The majority of intervention keywords in our taxonomy will have one of two parents: (1) DALY gross interventions, or (2) QALY gross interventions. There are two exceptions to this structure, which necessitate adding a lower level to the taxonomy. These include the following:
   1. Polyhierarchy structure: For interventions that were shared across causes, we used a polyhierarchical approach wherein the same keyword had multiple parents (**Figure S1.1**). This was limited to chemotherapies and immunotherapies where the same drug classes were used to treat multiple causes, as well as imaging techniques that were used to screen for a variety of conditions. For example, “alkylating antineoplastic agents” had several parent keywords, such as “chemotherapy for biliary tract cancer” and “chemotherapy for B-cell lymphoma.”
   2. Variations on the level of detail provided for a given intervention: We added lower levels to the taxonomy when interventions were described using varying levels of detail in order to retain information on these distinctions (**Figure S2.1**). In these instances, every ratio would be mapped to the parent, and only those ratios with more detail would also be mapped to the lower level. For example, every ratio that involves antiplatelet therapy is mapped to the intervention keyword “antiplatelet therapy.” In addition, when articles specify the drugs they are evaluating, their corresponding ratios are also mapped to the drug classes for these drugs (e.g. “antiplatelet therapy with COX inhibitors).
4. If a particular intervention is used across multiple causes and the specific intervention did not differ by cause, we did not include the cause name in the intervention keyword. On the other hand, when the same category of intervention is across causes, yet the cause has a strong influence on the specifics of the intervention (e.g. dosage, timing, frequency), we included the cause name in the intervention keyword.


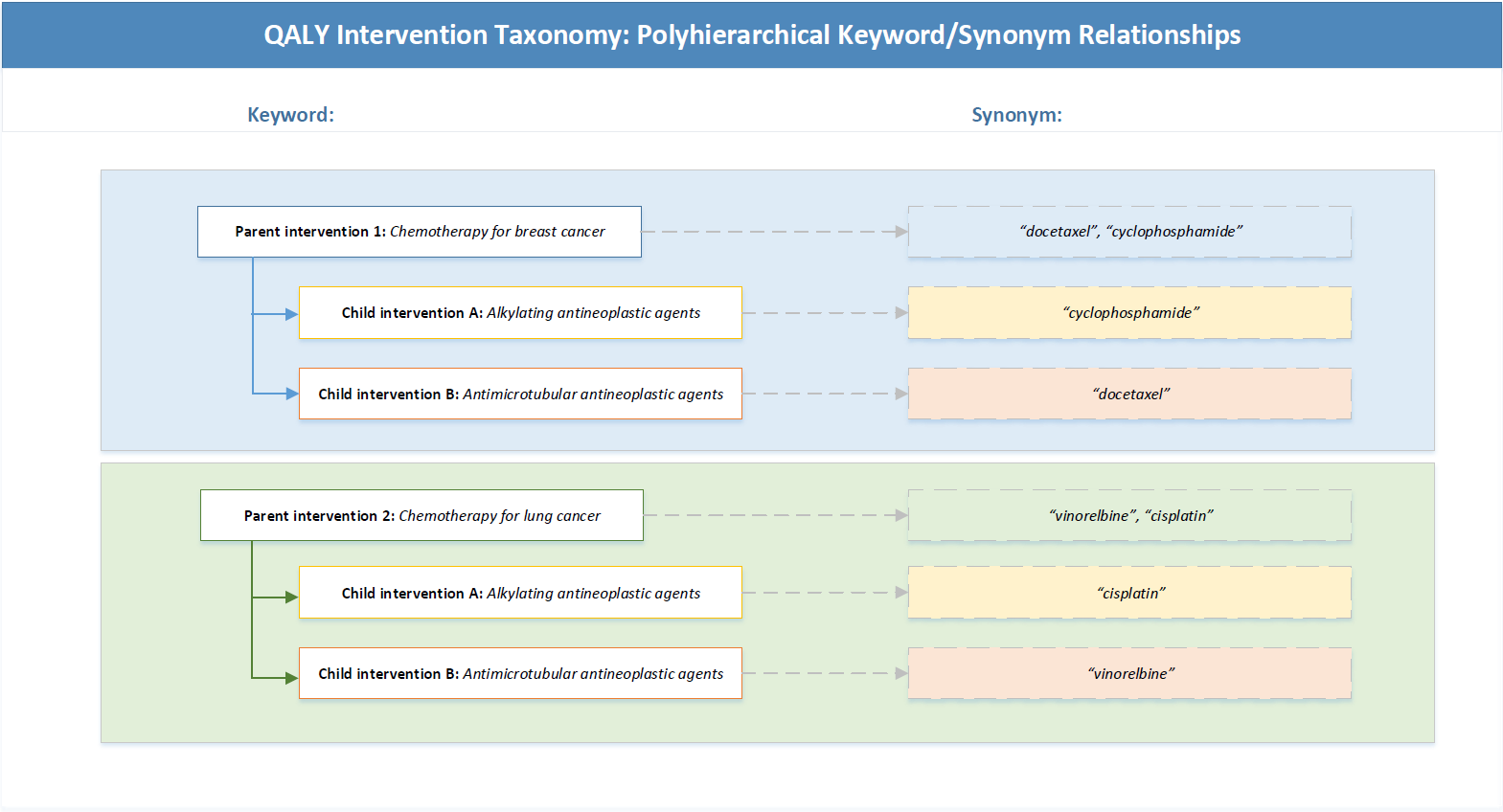


**S1.1 Figure. QALY Intervention Taxonomy: Polyhierarchical keyword/synonym relationships**

Polyhierarchical relationships in intervention taxonomy. Each parent intervention keyword has child keywords that represent specific drug classes for each type of chemotherapy. These drug classes are used to treat multiple cancer types, and thus have multiple parents. For example, “Alkylating antineoplastic agents” is a child with parents “Chemotherapy for breast cancer” and “Chemotherapy for lung cancer.” Therefore, interventions descriptions in the Tufts registries that contain the word “cisplatin” will be mapped to “Child intervention A” and “Parent intervention 2.” This allows us to meta-regress cost-effectiveness ratios for all chemotherapy interventions for a particular type of cancer (e.g. chemotherapy for breast cancer), or to meta-regress cost-effectiveness ratios across cancer types for a particular drug class (e.g. alkylating antineoplastic agents).


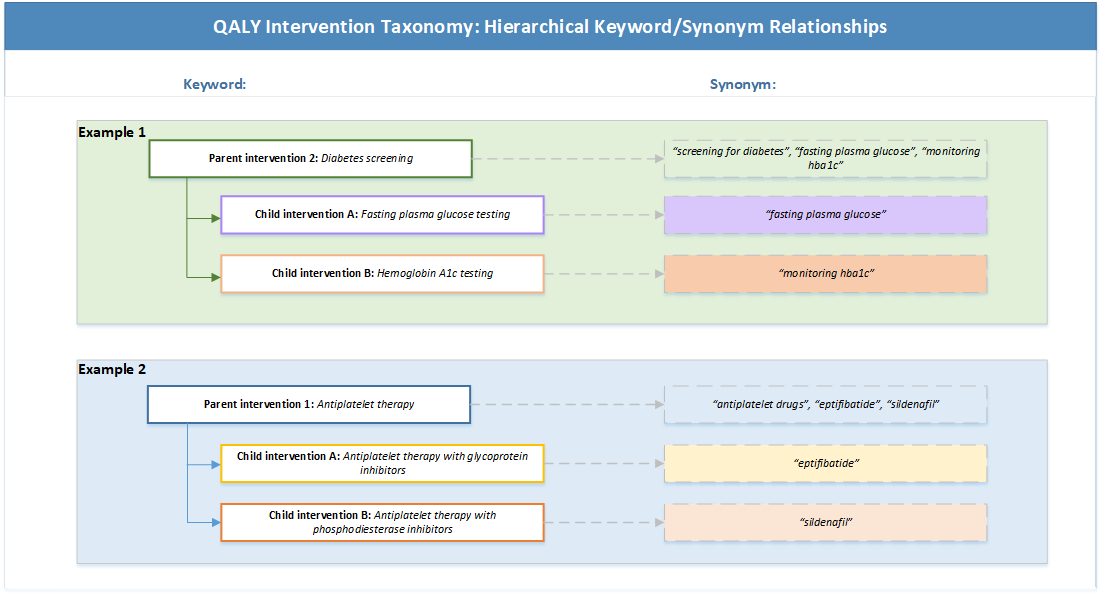


**S1.2 Figure.** **QALY Intervention Taxonomy: Hierarchical keyword/synonym relationships**

Legend: Hierarchical relationships in intervention taxonomy to accommodate varying levels of intervention detail. Articles describe interventions with varying levels of detail. In order to retain as much data as possible, we create parent-child relationships that allow for different degrees of intervention specificity. For example, when articles only describe interventions as “diabetes screening” without reference to the specific type of screening, we include synonyms for the parent “Diabetes screening” only. Interventions that include fasting plasma glucose testing or hemoglobin A1c testing are mapped to the parent and the more specific child. This allows us to run a regression for the parent intervention (e.g. all diabetes screening interventions), or to filter down to specific types of screening (e.g. all fasting plasma glucose testing interventions).

##

**S1 Appendix References**

1. Schulz R, Czaja SJ, McKay JR, Ory MG, Belle SH. Intervention Taxonomy (ITAX): Describing Essential Features of Interventions (HMC). Am J Health Behav. 2010;34: 811–21.

2. Cochrane Collaboration. Effective Practice and Organization of Care (EPOC) Taxonomy. 2015 [cited 2021 Oct 24]. In: Cochrane EPOC Scope of Work [Internet] Available from: https://epoc.cochrane.org/epoc-taxonomy

3. OpenRefine Contributors. OpenRefine. [cited 2021 Oct 24]. In: OpenRefine/Download [Internet]. Available from: http://openrefine.org.
