# Supplementary material for: Cost-effectiveness of HPV vaccination in 195 countries: A meta-regression analysis
Source: PLoS One. 2021 Dec 20;16(12):e0260808. doi: 10.1371/journal.pone.0260808 (PMC8687557; doi:10.1371/journal.pone.0260808)
Supplement: S2 Appendix — (DOCX) [file pone.0260808.s004.docx]

**S2 Appendix. Data extractions and mapping**

**Section S2.1: Null comparator**

The Tufts registries include a categorical variable for the comparator for each ICER, including none, placebo, standard of care, or other. They also included a text variable that described the comparator in more detail. When these data were missing, we returned to the articles to extract the comparator description and map the comparator to one of the aforementioned four categories.

When possible, we re-calculated ICERs compared to “other” such that they were compared to “null”. For articles that did not report sufficient data to re-calculate ICERs relative to no intervention, we re-calculated them compared to either “placebo” or “standard of care”. We used data in the Tufts registries on total or per person costs and total or per person health benefits. For ratios without these data reported in the registries, we went back to the articles to extract the necessary data to re-calculate the ICERs relative to the null comparator (i.e. “none”, “placebo”, or “standard of care”). We removed ratios that could not be re-calculated to the null comparator, as well as ratios that were compared to “other” if we had another ratio for the same intervention compared to the null.

We defined the null comparator as no intervention, standard of care, or placebo. An exception was studies where the standard of care was screening for HPV infection; we defined a variable, “screening comparator” to estimate the effect of this higher standard of care for cervical cancer prevention.

**Section S2.2: Time horizon**

For ratios missing the time horizon variables in the Tufts registries, we returned to the papers to extract the time horizon magnitude and units (weeks, months, years, lifetime). For our analysis, we created a dichotomous variable: lifetime vs. not lifetime. If the article did not clearly state the time horizon, but the authors explicitly cite including mortality as an outcome of their analysis, we coded the corresponding ratios as having a lifetime time horizon. We removed articles from our analysis that either did not clearly state the time horizon in the article, or did not clearly state mortality as an outcome of their analysis.

**Section S2.3: Discount rate**

The Tufts registries include variables that capture the discount rate for costs and health outcomes (QALYs or DALYs). For those ratios missing discount rate data, we returned to the papers to extract these values. When articles referenced standard methods, we extracted the discount rates cited in those methods (e.g. World Health Organization (WHO) Choosing Interventions that are Cost-Effective (CHOICE). We removed ratios from our analysis if the discount rates were not clearly stated in the article.

**Section S2.4: Age**

The Tufts registries include several categorical variables for age. When these data were available, we mapped the Tufts age variables to GBD age variables. For those ratios with missing age information in the Tufts data (52% of all ratios), we used the abstract, target population description, or returned to the articles to extract the age information. If there was no description of the age in the article, we defaulted to the GBD age groups used for modeling the cause(s) targeted by that particular intervention. We made the following additional assumptions when the age was not explicitly stated in the article: (1) policies, laws, and infrastructural interventions affect all ages; (2) interventions targeting pregnant women were mapped to GBD’s definition for reproductive-aged women (ages 15-49 years); (3) interventions targeting adults were assumed target individuals ages 15 and older. Finally, we adjusted all age-mapping such that the age variable captured the beneficiary target population in instances where the target population and beneficiary population differed. These primarily including prevention of mother to child transmission interventions where the mother is targeted and the child benefits, as well as HPV vaccines, where the target age is in adolescence, but the health benefits with respect to cancer prevention are often not realized until later in life.

**Section S2.5: Sex**

For ratios missing sex information, we created a Python dictionary to map interventions to male, female, or both. This dictionary included causes that are sex-specific (e.g. prostate cancer, ovarian cancer), as well as other sex-specific interventions (e.g. male circumcision, mammography, tubal ligation). For ratios that were not mapped using the Python dictionary approach, we used the Tufts target population descriptions to map ratios to sex.

**Section S2.6: Causes**

In the Tufts registries, articles are mapped to one or more causes or risk factors, yet neither registry used the most recent GBD 2017 cause hierarchy. We needed each ratio to be mapped to one or more GBD 2017 causes, etiologies, or impairments in order to leverage the most recent GBD data. We first developed a mapping of Tufts causes to GBD 2017 causes. For articles with only one cause, we mapped all of the ratios in that article to that cause. For articles with multiple ratios, we first created a Python dictionary with cause-specific keywords to map each ratio to the most detailed cause(s) possible. Next, for those we could not map with the dictionary approach, we used Doctor Evidence software (<https://drevidence.com/>). We uploaded article titles to Doctor Evidence, which used a mapping algorithm to map keywords in those titles to ICD9 and ICD10 codes. We then used the ICD9 and ICD10 to GBD cause mapping algorithm to map these articles to GBD causes. Because these two processes (dictionary mapping and DRE) were automated, we validated them by reviewing the causes each ratio was mapped to and made changes to an erroneous mappings. For the remaining ratios that we were unable to map through the processes outlined above, we used the abstract or returned to the articles to map each of these ratios to GBD causes. Finally, we removed any ratios that could not be mapped to GBD causes.

**Section S2.7: Locations**

The Tufts registries included a text variable for the target population country of the intervention. We merged this variable with the GBD location hierarchy. For locations that did not readily merge with GBD locations, we manually mapped them. For ratios that were mapped to regions or super-regions rather than countries, such as a series of WHO CHOICE articles,[2] we returned to the articles to extract country-level ratios if they were present. If there were no country-level results, we excluded these articles.

**Section S2.8: Delivery platforms**

We mapped each ratio to one or more delivery platforms. We adapted the delivery platforms outlined in an article by Jamison and colleagues.[2] These included the following platforms: (1) intersectoral policies to reduce behavioral and environmental risks; (2) population-based; (3) community; (4) health facility; (5) first-level hospital; and (6) referral and specialty hospital. In addition, we separated the referral and specialty hospital platform into inpatient and outpatient, due to the differences in healthcare personal and infrastructure required for inpatient and outpatient services. When the delivery platforms were not clearly stated in the intervention descriptions or abstracts of the articles within the Tufts registries, we returned to the articles to determine the delivery platform. When the articles did not include delivery platform information, we Jamison et al.’s categorization of interventions as guidance.^2^ For interventions that were not represented in Jamison et al., we relied on domain knowledge for each intervention to map interventions to delivery platforms. Because the majority of HPV vaccine interventions were delivered at health facilities, we had the most data for this platform, and thus restricted this analysis to the health facility platform.

Definitions for the delivery platforms outlined by Jamison and colleagues and adapted for our analysis are provided below with examples.

Platform 1: Intersectoral policies to reduce behavioral and environmental risks. All policies outside of health sector

*Examples: (1) taxes on alcohol/tobacco; (2) trans-fat ban; (3) salt regulations with industry; (4) public transit; (5) traffic safety*

Platform 2: Population-based. This platform captures all non-personal or population-based health services, such as mass media and social marketing of educational messages, typically delivered by public health agencies.

*Examples: (1) mass media messages concerning awareness on handwashing; (2) sustained vector management for Chagas disease; (3) decentralize stocks of antiviral medications to improve access for at-risk populations; (4) ensure influenza vaccine security at national and sub-national level; (5) community-based HIV testing; (6) WASH behavior-change interventions (e.g. community-led sanitation)*

Platform 3: Community. The community platform encompasses efforts to bring health care services to clients, meeting people where they live. It includes a wide variety of delivery mechanisms. Specific sub platforms include: health outreach and campaigns ( e.g. vaccination campaigns, mass deworming, and face-to-face education and communication); schools (including school health days); and community health workers, who may be based primarily in the community but also connected to first-level care providers with ties to the rest of the system.

*Examples: (1) ANC and postpartum education on family planning; (2) counseling mothers on newborn care; (3) Childhood vaccines (rotavirus, pneumococcus, measles, hep B); (4) education on handwashing and safe disposal of children's stools*

Platform 4: Health center. The health center level captures two types of facility. The first is higher-capacity health facility staffed by a physician or clinical officer and often with a midwife to provide basic medical care, minor surgery, family planning and pregnancy services, and safe childbirth for uncomplicated deliveries. The second is a lower-capacity facility (e.g. health clinics, pharmacies, dental offices) staffed primarily by a nurse or mid-level health care provider, providing services in less-resourced and often more remote settings.

*Examples: (1) early detection and treatment of neonatal pneumonia; (2) hypertension screening; (3) PMTCT of HIV and syphilis; (4) partner notification and treatment for common STIs, including HIV; (5) PREP for HIV; (6) HIV screening in all individuals with TB; (7) provision os aspirin for all cases of suspected acute MI; (8) tobacco cessation counseling; (9) screening and intervention for alcohol use disorders*

Platform 5: First-level hospital. A first-level hospital is a facility with the capacity to perform surgery and provide inpatient care. This platform also includes outpatient specialist care and routine pathology services that cannot be feasibly delivered at lower levels, such as newborn screening.

*Examples: (1) induction of labor post-term; (2) surgery for ectopic pregnancy; (3) tubal ligation; (4) calcium and vitamin D supplementation for osteoporosis secondary prevention; (5) medical management of acute heart failure; (6) hernia repair; (7) appendectomy; (8) basic skin grafting; (9) assisted vaginal delivery using vacuum extraction or forceps*

Platform 6: Referral and specialty hospital outpatient. This platform includes general specialists that provide secondary and tertiary services in an outpatient setting.

*Examples: (1) specialized TB services (managing MDR and XDR TB); (2) treatment of early stage cancers*

Platform 7: Referral and specialty hospital inpatient. This platform includes general specialists that provide secondary and tertiary services in an inpatient setting.

*Examples: (1) full supportive care for preterm newborns; (2) repair of cleft lip and palate*

**S2 Appendix References**

1. Evans DB, Adam T, Edejer TT-T, Lim SS, Cassels A, Evans TG. Time to reassess strategies for improving health in developing countries. BMJ 2005; 331: 1133–6.

2. Jamison DT, Alwan A, Mock CN, Nugent R, Watkins D, Adeyi O, et al. Universal health coverage and intersectoral action for health: key messages from Disease Control Priorities, 3rd edition. Lancet. 2018;391: 1108–20.
