# Supplementary material for: Cost-effectiveness of HPV vaccination in 195 countries: A meta-regression analysis
Source: PLoS One. 2021 Dec 20;16(12):e0260808. doi: 10.1371/journal.pone.0260808 (PMC8687557; doi:10.1371/journal.pone.0260808)
Supplement: S3 Appendix — (PDF) [file pone.0260808.s005.pdf]

S3 Appendix. Meta-regression analysis

This appendix details the statistical model and fitting procedure used to obtain the estimated curves and posterior uncertainty presented in the paper. For technical details on model fitting please see [11].

The analysis is broken up into five stages. First, we conduct crosswalk analyses of four covariates in order to leverage the sensitivity analyses reported in the studies from the Tufts registries, as described in Section S3.1.

In the second stage, we estimate a nonlinear response curve for log-GDP per capita, as detailed in Section S3.2. This analysis uses splines to represent the curve, nonlinear observation models for relative risks, a robust statistical approach for outlier detection, and a spline ensemble to make the model less sensitive to model specification. Grouping is ignored in this stage of the analysis.

In the third stage, we use the nonlinear response curve estimated in the second stage to select potential bias covariates, using a generalized Lasso approach for linear mixed effects models, detailed in Section S3.3.

In the fourth stage, as described in Section S3.4, we use 10-fold cross-validation to select the standard deviation of a Gaussian prior to apply to all covariates other than those analyzed in the first stage.

In the fifth stage, we include covariates that were detected in the third stage, along with the nonlinear response, and consider a mixed effects model with a random intercept, as discussed in Section S3.5.

S3.1. Crosswalk analyses of sensitivity analysis covariates

Univariate sensitivity analyses are used to estimate the effect of a variable on the ICER with all other variables held constant by definition. We analyzed the difference in log-ICERs between sensitivity analyses and the corresponding reference analyses using models which we refer to as crosswalks. Including the results of these models as priors in subsequent steps of the analysis decreases omitted variable bias by giving more influence to pairs of ICERs which we know differ in no unmeasured variables. It also stabilizes estimates in the presence of multicollinearity. See the correlation matrix in Table S3.1 below.

We conducted crosswalk analyses for four variables, vaccine cost, cost discount rate, DALY/QALY discount rate, and coverage. These covariates had a sufficient number of sensitivity analyses for us to control for study-specific variables not included in our model by using comparisons of sensitivity analyses and main results. For each of these covariates, we paired each sensitivity analysis with another ICER from the same study and location and which differed only in that covariate. For these covariates, we fit separate models of the form

$$y_{i,sens} - y_{i,ref} = \alpha_c \times (x_{c_{i,sens}} - x_{c_{i,ref}}) + \epsilon_i \tag{1}$$

Where x_c is the only covariate that differs between the two analyses, $y_{i,sens}$ and $y_{i,ref}$ are the log-ICERs of sensitivity analysis i and its corresponding reference analysis, and $x_{c_{i,sens}}$ and $x_{c_{i,ref}}$ are the respective values of x_c. $\epsilon_i \sim N(0, \hat{\sigma}_c^2)$ are independent errors.

The resulting coefficients, $\hat{\alpha}_c$, their standard errors, $\widehat{SE}[\hat{\alpha}_c]$ and sample size for the estimates are reported in Table S3.2. Note that some sensitivity analyses were used in more than one crosswalk model. We use the coefficients and their standard errors as Gaussian priors in the models run in all subsequent stages of the analysis.

	log-ICER	log GDP per capita	log Cervical cancer DALYs per capita	log Vaccine Cost	Cost Discount Rate	DALY/QALY Discount Rate	Coverage	Vaccine Type	Burden Measure	Payer Perspective	Screen Comparator	Limited Time Horizon	Access to Care 100%	No Booster
log-ICER	1.0													
log GDP per capita	0.7	1.0												
log Cervical cancer DALYs per capita	-0.59	-0.66	1.0											
log Vaccine Cost	0.86	0.72	-0.45	1.0										
Cost Discount Rate	0.11	0.01	-0.01	0.01	1.0									
DALY/QALY Discount Rate	0.13	-0.07	0.06	-0.09	0.45	1.0								
Coverage	0.25	0.23	-0.13	0.33	-0.07	-0.05	1.0							
Vaccine Type	0.36	0.41	-0.26	0.39	-0.05	0.01	-0.09	1.0						
Burden Measure	0.54	0.64	-0.44	0.62	-0.06	-0.06	0.1	0.73	1.0					
Payer Perspective	0.11	0.17	-0.09	0.21	-0.03	-0.01	0.37	0.14	0.29	1.0				
Screen Comparator	0.26	0.31	-0.04	0.37	0.01	-0.07	0.02	0.19	0.23	-0.05	1.0			
Lifetime Horizon	-0.26	-0.28	0.19	-0.26	0.07	0.08	0.02	-0.13	-0.32	-0.18	-0.14	1.0		
Access to Care 100%	-0.54	-0.56	0.42	-0.59	0.01	0.05	-0.41	-0.51	-0.66	-0.31	-0.15	0.21	1.0	
Vaccine Sex	-0.14	-0.12	0.07	-0.2	-0.04	0.04	-0.18	0.15	0.06	0.21	-0.1	0.01	0.08	1.0
No Booster	-0.24	-0.28	0.19	-0.22	-0.01	-0.03	0.02	-0.34	-0.33	-0.17	-0.11	0.36	0.23	-0.02

Table S3.1. Covariate Correlation Matrix

Covariate	$\hat{\alpha}_c$	$\widehat{SE}[\hat{\alpha}_c]$	Sample Size
log-Vaccine Cost	1.073	0.006	824
Cost Discount Rate	0.150	0.016	78
DALY/QALY Discount Rate	0.510	0.009	122
Coverage	0.0125	0.00107	111

Table S3.2. Parameter estimates for crosswalk analyses

S3.2. Estimation of Nonlinear log-GDP per capita response curve

The relationship between log-ICER and log-GDP per capita is modeled using a basis spline (B-spline) [3, 4]. In this section, we present B-splines, specification of constraints, discussion of trimming, and summary of spline ensembles. Portions of Section S3.2 have been reproduced or adapted from GBD Risk Factor Collaborators [6].

S3.2.1. B-splines linear tails

A spline basis is a set of piecewise polynomial functions with designated degree and domain. If we denote polynomial order by p, and the number of knots by k, we need $p + k$ basis elements s_j^p, which can be generated recursively as illustrated in Figure S3.1.

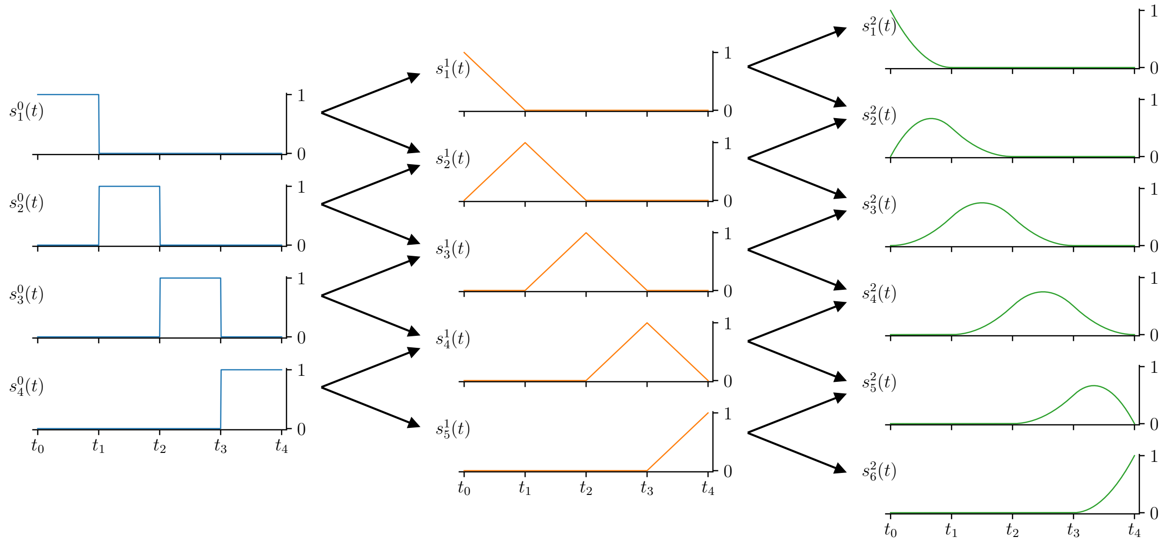

Figure S3.1. Recursive generation of bspline basis elements (orders 0, 1, 2).

Given such a basis, we can represent any curvilinear relationship as the linear combination of the spline basis elements, with coefficients $\boldsymbol{\beta} \in \mathbb{R}^{p+k}$:

$$f(t) = \sum_{j=1}^{p+k} \beta_j^p s_j^p(t). \tag{2}$$

An explicit representation of (2) is obtained by building a design matrix $\mathbf{X}$. Given a set of t values at which we have data, the jth column of $\mathbf{X}$ is given by the expression

$$\mathbf{X}_{\cdot,j} = \begin{bmatrix} s_j^p(t_0) \\ \vdots \\ s_j^p(t_k) \end{bmatrix}. \tag{3}$$

For extreme values of log-GDP per capita with little data, we need the capability to ensure that the outermost segments of the spline are linear, with slopes that match the adjacent segment at the knot. Splines with linear tails are often called *natural* splines.

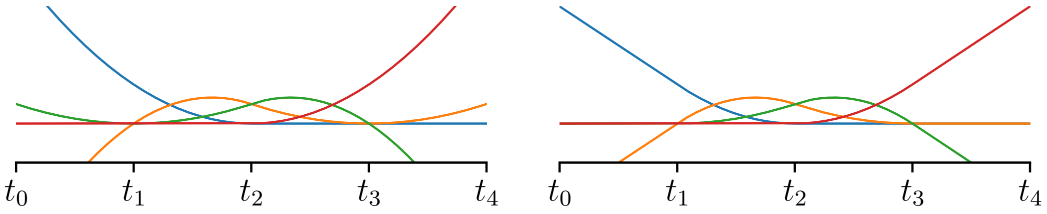

Figure S3.2. Right: spline bases. Right: spline bases with linear tails.

S3.2.2. Robust Trimming Strategy

To robustify the approach against outliers, we use the trimming strategy, as discussed in [1, 11]. The estimator

$$\min_{\boldsymbol{\beta} \in \boldsymbol{B}} \sum_i f_i(\boldsymbol{\beta}),$$

where f_i is as in (2) and $\boldsymbol{B}$ encodes all necessary constraints is extended to the 'trimmed' estimator

$$\min_{\boldsymbol{w} \in \Delta,\ \boldsymbol{\beta} \in \boldsymbol{B}} \sum_i w_i f_i(\boldsymbol{\beta})$$

where each w_i is required to be between 0 and 1, and the total mass of w is constrained to equal 90% of the data volume. Specifically, this means that

$$\Delta = \{w : 0 \leq w_i \leq 1, \sum w_i = 0.9N\}$$

where N is the total number of data points across studies. Thus the trimmed estimator finds the 90% most fittable data and fits them for $\boldsymbol{\beta}$. Selecting a proportion of trimming has had a long history in terms of theory [9] and recent methodological innovations [1]. However, thus far it has not been possible to automatically select the expected number of inliers. We chose 90% in order to include the vast majority of the data while remaining robust to a potential set of outliers. The same choice has been made in larger systematic analyses [6] as well. Alternative proportions are available, but we did not experiment with them.

S3.2.3. Spline ensemble

Every model estimate intrinsically depends on the choice of knot placement used to generate the spline. To remove the effect of this choice on the estimates, we develop an ensemble over this knot placement, leaving only the choice of spline degree and number of knots as modeling choices.

Given the degree and number of knots, we automatically sample a set of knot placements for a feasible knot distribution. For each resulting knot placement, we fit a spline (using the trimming estimator) and then evaluate each resulting model by computing its fit and curvature, aggregating the final model as a weighted combination of the ensemble.

S3.2.4. Sampling Knots From Simplex

To establish a reasonable feasible set from which to sample, we prefix a minimal set of the rules for the knot-placement and uniformly sample from this feasible set. Given a number of knots, the rules specify feasible ranges for each knot, and feasible gaps between knots. Specifically, given an interval $[t_0, t_k]$

delimited by terminal knots (which are always the minimum and maximum of the data), the feasible region of the interior knots $t_1, \ldots, t_{k-1}$ is given by

$$t_i \in [a_i, b_i], \quad \text{for} \quad i = 1, \ldots, k-1, \qquad t_i - t_{i-1} \in [c_i, d_i] \quad \text{for} \quad i = 1, \ldots, k.$$

We enforce the rules

$$a_i \geq t_0, \quad b_i \leq t_k, \quad c_i \geq 0, \quad \sum c_i \leq t_k - t_0.$$

The knot placement that satisfy these four rules comprise a closed polyhedron $\{\boldsymbol{t} : \mathbf{P}\boldsymbol{t} \leq \boldsymbol{p}\}$, where,

$$\mathbf{P} = \begin{bmatrix} \mathbf{I} \\ -\mathbf{I} \\ \mathbf{D} \\ -\mathbf{D} \end{bmatrix}, \quad \boldsymbol{p} = \begin{bmatrix} \boldsymbol{b} \\ -\boldsymbol{a} \\ \boldsymbol{d} \\ -\boldsymbol{c} \end{bmatrix}, \quad \mathbf{D} = \begin{bmatrix} -1 & 1 & & \\ & \ddots & \ddots & \\ & & -1 & 1 \end{bmatrix}, \quad \boldsymbol{b} = \begin{bmatrix} b_1 \\ \vdots \\ b_{k-1} \end{bmatrix}, \quad \boldsymbol{c} = \begin{bmatrix} c_1 \\ \vdots \\ c_k \end{bmatrix}, \quad \boldsymbol{d} = \begin{bmatrix} d_1 \\ \vdots \\ d_k \end{bmatrix}$$

We calculate the vertices of the polyhedron using the *double description method* in [7], and uniformly sample knot-placements from within the polyhedron. Each knot placement yields a model, fit using the trimmed constrained spline approach described above.

S3.2.5. Scoring

Once the ensemble is created, we score the resulting risk curves using two criteria: model fit (measured using the log-likelihood) and total variation (measured using the highest order derivative). These scores balance competing objectives of fit and generalizability. Once we have these scores, denoted as $\boldsymbol{s}_1$ and $\boldsymbol{s}_2$, we normalize them to the range $[0, 1]$:

$$\boldsymbol{v}_i = \frac{\boldsymbol{s}_i - \min(\boldsymbol{s}_i)}{\max(\boldsymbol{s}_i) - \min(\boldsymbol{s}_i)}$$

and apply a logistic transformation. The transformation is used to make the scoring meaningful even in the presence of spurious curves in a large ensemble. We then multiply the scores

$$\boldsymbol{w} = \boldsymbol{w}_1^{p_1} \odot \boldsymbol{w}_2^{p_2}.$$

to down-weight models that are low under either criterion (fit or total variation). The final weights are normalized to sum to 1.

S3.2.6. New nonlinear `signal` covariate

We fit a model of log-ICER on log-GDP per capita using a robust spine ensemble on log-GDP per capita with degree 2, two knots, and linear tails. This model also includes as covariates log cervical cancer DALYs per capita and the four crosswalk covariates. We placed Gaussian priors with means $\hat{\alpha}_c$ and standard deviation $\widehat{SE}[\hat{\alpha}_c]$ on the crosswalk covariates' coefficients. We used this model to generate a nonlinear log-GDP per capita response curve, which is encoded into a new nonlinear covariate called '`signal`' and included in subsequent stages of the analysis. The shape of this transformation is displayed in Figure S3.3.

In particular, this allows us to fit a linear mixed effects model of the form

$$y_{ij} = \beta_0 + \texttt{signal}_{ij} \times (\beta_s) + x_{1_{ij}}\beta_1 + \ldots + x_{k_{ij}}\beta_k + \epsilon_{ij} + u_i \tag{4}$$

where $\epsilon_{ij} \sim N(0, \sigma_{ij}^2)$ are known each observation, and $u_i \sim N(0, \gamma)$ is a random study-specific intercept, with unknown variance γ.

S3.3. Covariate selection using Lasso

Additional covariates are selected using a Lasso stategy described below in the context of linear mixed effects models [2][8]. In considering potential covariates, we enforce that every categorical covariate has some variation; in particular every indicator covariate has at least two studies in each category.

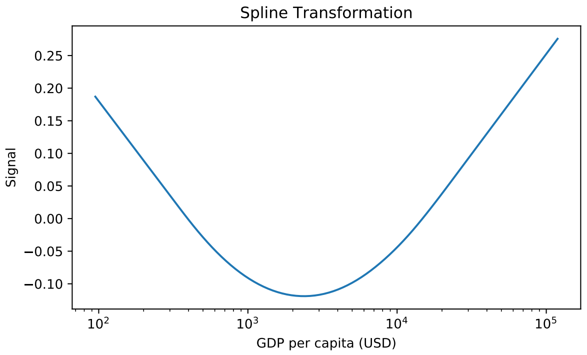

Figure S3.3. Nonlinear transformation of the log GDP per capita variable.

- We iteratively decrease the weight on the Lasso regularizer and let coefficients of bias-covariates enter the model in the order derived from the Lasso solutions.
- As a group of coefficients enters the model, we test it for statistical significance.
  - If the coefficients are significant, we compute their posterior distribution and use this posterior as the prior for these coefficients for the next round.
  - If the coefficients are not significant, the process terminates, and we return the list of (significant) covariates obtained so far.

We included the `signal` and the four crosswalk covariates as pre-selected covariates without the Lasso regularizer in all models above. We added Gaussian priors to the coefficients of the crosswalk covariates with mean $\hat{\alpha}_c$ and standard deviation $\widehat{SE}[\hat{\alpha}_c]$, as estimated in Section S3.1.

Covariates with low variance or that are highly correlated with others are unlikely to be selected by this process, since including them would likely inflate the variance of the resulting estimators by an amount that outweighs the reduction in bias. This is a limitation of the current data set, and future work to expand the data set by extracting sensitivity analyses for a wider number of covariates could allow for the stable estimation of additional parameters.

There is ongoing methodological work to improve variable selection in the presence of collinearity. Based on early work showing the advantages of bridge regression vs. lasso [5] in the presence of correlation, the elastic net penalty [12] has been used, and in principle able to find groups of correlated predictors. Practical use requires additional parameter selection. We are currently looking into methods based on nonconvex regularizes as well [10]. These innovations can further improve variable selection in future work, but now we test for collinearity using basic tests before the lasso procedure starts.

One of the difficult questions in any variable selection procedure is when to stop. The methodology in step 1 builds on the Lasso methodology, but has an automatic termination criteria, stopping as soon as sequentially selected variables (selected across a range of the Lasso parameter) cease to be statistically significant in a standard Gaussian analytical framework.

Bias covariates that pass the selection process are included in the next stage of the model fitting.

S3.4. Gaussian prior cross validation

In order to further safeguard against overfitting, we included a Gaussian prior on all covariates. We used 10-fold cross validation to determine the prior standard deviation, τ_{cv}, to apply to the coefficients of all covariates other than the four crosswalk covariates. We fit models of the same form as (4) with priors on coefficients β. For the four crosswalk covariates, we used the priors calculated in Section S3.1.

Covariate	$\hat{\beta}$	$\widehat{SE}[\hat{\beta}]$	$\hat{\gamma}$
Intercept	-1.02	0.14	0.51
`signal`	0.51	0.09	
log Vaccine Cost	1.06	0.005	
Coverage	0.006	0.001	
Cost Discount Rate	0.003	0.008	
DALY/QALY Discount Rate	0.49	0.001	
log Cervical cancer DALYs per capita	-0.39	0.01	
Limited Time Horizon	0.02	0.08	
Vaccine Type	-0.17	0.03	
Screen Comparator	-0.03	0.04	
Access to Care 100%	-0.003	0.037	
Burden Measure	-0.006	0.0422	
Vaccine sex	0.67	0.04	
	R^2	RMSE	Sample Size
Sample with sensitivity analyses			1522
Fixed and Random Effects	0.94	0.53	
Fixed Effects only	0.89	0.70	
Sample of CE registry entries only			471
Fixed and Random Effects	0.95	0.55	
Fixed Effects only	0.92	0.71	

Table S3.3. Parameter estimates for the final model

For all others, we used a common $N(0, \tau_{cv}^2)$ prior on their coefficients after standardizing the covariates to have mean 0 and unit variance. We used a grid-search to select the value of τ_{cv}, that minimizes the MSE for predicting data in the hold-out set.

S3.5. Meta-Regression Analysis

Once the `signal` covariate is obtained (Section S3.2), bias covariates are selected (Section S3.3), and priors are calculated for crosswalk (Section S3.1) and non-crosswalk (Section S3.4) covariates, we convert the priors on standardized covariates calculated in Section S3.4 to an unstandardized scale and fit a final model of the form

$$y_{i,j} = \beta_0 + \beta_1 x_{1_{i,j}} + ... + \beta_k x_{k_{i,j}} + \epsilon_{i,j} + u_j \tag{5}$$

where coefficients for crosswalk covariates have priors $\beta_c \sim N(\hat{\alpha}_c, \widehat{SE}[\hat{\alpha}_c]^2)$, as estimated in Section S3.1. Coefficients for all other covariates selected in Section S3.3, including the `signal` covariate, have priors $\beta_l \sim N(0, \tau_{cv}^2)$, as selected in Section S3.4. $u_j \sim N(0, \gamma)$ is a random study-specific intercept and $\epsilon_{i,j} \sim N(0, \sigma_\epsilon^2)$ are independent error terms. Parameter estimates are displayed in Table S3.3.

Parameters β and γ are obtained using maximum likelihood, as detailed in [11]. Standard errors of β are estimated by taking the standard deviation across 1000 samples from the posterior distribution of $\hat{\beta}$.

References

[1] A. Aravkin and D. Davis. Trimmed statistical estimation via variance reduction. *Mathematics of Operations Research*, 2019.

[2] H. D. Bondell, A. Krishna, and S. K. Ghosh. Joint variable selection for fixed and random effects in linear mixed-effects models. *Biometrics*, 66(4):1069–1077, 2010.

[3] C. De Boor, C. De Boor, E.-U. Mathématicien, C. De Boor, and C. De Boor. *A practical guide to splines*, volume 27. springer-verlag New York, 1978.

[4] J. H. Friedman et al. Multivariate adaptive regression splines. *The annals of statistics*, 19(1):1–67, 1991.

[5] W. J. Fu. Penalized regressions: The bridge versus the lasso. *Journal of computational and graphical statistics*, 7(3):397–416, 1998.

[6] GBD 2019 Risk Factors Collaborators. Global burden of 87 risk factors in 204 countries and territories, 1990–2019: a systematic analysis for the global burden of disease study 2019. *The Lancet*, 396(10258):1223–1249, 2020.

[7] T. S. Motzkin, H. Raiffa, G. L. Thompson, and R. M. Thrall. The double description method. *Contributions to the Theory of Games*, 2(28):51–73, 1953.

[8] S. Müller, J. L. Scealy, and A. H. Welsh. Model selection in linear mixed models. *Statistical science*, 28(2):135–167, 2013.

[9] P. J. Rousseeuw. Multivariate Estimation with High Breakdown Point. *Mathematical statistics and applications*, 8:283–297, 1985.

[10] P. Zheng, T. Askham, S. L. Brunton, J. N. Kutz, and A. Y. Aravkin. A unified framework for sparse relaxed regularized regression: Sr3. *IEEE Access*, 7:1404–1423, 2019.

[11] P. Zheng, R. Barber, R. Sorensen, C. Murray, and A. Aravkin. Trimmed constrained mixed effects models: Formulations and algorithms. *Journal of Computational and Graphical Statistics*, 30:1–13, 2021.

[12] H. Zou and T. Hastie. Regularization and variable selection via the elastic net. *Journal of the Royal Statistical Society. Series B, Statistical methodology*, 67(2):301–320, 2005.
