# Supplementary material for: Cost-effectiveness of HPV vaccination in 195 countries: A meta-regression analysis
Source: PLoS One. 2021 Dec 20;16(12):e0260808. doi: 10.1371/journal.pone.0260808 (PMC8687557; doi:10.1371/journal.pone.0260808)
Supplement: S4 Appendix — (DOCX) [file pone.0260808.s006.docx]

**S4 Appendix. Cost-saving predictions**

We built a logistic regression model with both fixed and random effects to predict the probability of an HPV intervention being cost saving. In this analysis, one meant that the cost-effectiveness result was cost saving with a decrease in cost and increase in DALYs or QALYs averted, and zero meant that the result was in the ICER quadrant with an increase in cost and increase in DALYs or QALYs averted. The model was trained on data from the Tufts registries; 1828 ratios from 75 articles, of which 136 ratios (7.4%) from 25 articles were cost-saving. In order to account for between study heterogeneity, ratios were grouped by article and a random intercept was calculated for each article.

Initially, we built a model that included the same set of covariates as the meta-regression analysis. These fixed covariates were: GDP per capita, cost of a full vaccine series (in 2017 USD), vaccine coverage, cost discount rate, discount rate for health outcomes, cervical cancer DALYs per person, time horizon (lifetime or less than lifetime), vaccine type (bivalent or quadrivalent), the comparator intervention (screening or no intervention), the proportion of the population with access to cancer treatment (100% or less than 100%), outcome measure (DALYs or QALYs), the target sex (female or both), and the perspective (payer or societal). We removed outcome measure due to collinearity with the access to care variable. We removed the comparator intervention, because of the large standard error around the parameter estimate. We log transformed GDP per capita, DALYs per person, and vaccine cost to correct for a right skewed distribution of both of these variables.

Results of the logistic regression model, including estimates of the coefficients for all covariates, along with standard error, Z value and p value are in **Table S4.1** Of all the covariates used in the model, log GDP per capita, log vaccine cost, cost discount rate, target sex, and log cervical cancer burden per capita were statistically significant.

| **Table S4.1. Logistic regression results for cost-saving predictions** | | | | |
| --- | --- | --- | --- | --- |
| **Variable** | **Estimate** | **Standard error** | **Z value** | **P value** |
| Intercept | 10.2 | 4.16 | 2.45 | 0.015 |
| Log GDP per capita (2017 USD) | 0.997 | 0.167 | 5.97 | <0.001 |
| Log vaccine cost (2017 USD) | -2.69 | 0.297 | -9.06 | <0.001 |
| Coverage (percentage) | -0.028 | 0.022 | -1.25 | 0.211 |
| Cost discount rate | -1.85 | 0.333 | -5.57 | <0.001 |
| DALY/QALY discount rate­ | 0.271 | 0.260 | 1.04 | 0.297 |
| Vaccine sex (ref = female) | -7.71 | 3.09 | -2.50 | 0.013 |
| Log cervical cancer DALYs per capita | 1.61 | 0.368 | 4.38 | <0.001 |
| Access to care (ref = < 100% access to care) | 0.325 | 3.73 | -0.087 | 0.931 |
| Vaccine type (ref = bivalent) | 1.48 | 1.81 | 0.816 | 0.412 |
| Perspective (ref = societal) | -2.65 | 2.35 | -1.13 | 0.260 |
| Time horizon (ref = lifetime) | 1.58 | 3.63 | 0.436 | 0.663 |

To evaluate model performance, we compared the model’s prediction of whether or not a given HPV vaccine intervention was cost saving to the training data from the Tufts registry. We used a threshold probability of 0.5 to classify the intervention as cost-saving. The model accuracy was 0.933, the sensitivity was 0.390 the specificity was 0.978, and the false positive rate was 0.022 (**Table S4.2**).

| **Table S4.2. Summary of logistic regression model performance using Tufts data** | | | |
| --- | --- | --- | --- |
| **Accuracy** | **Sensitivity** | **Specificity** | **False positive rate** |
| 0.933 | 0.390 | 0.978 | 0.022 |

We noted that the access to care variable was particularly unstable, because there was no variation in it within or between studies. Six of the published CEA assumed 100% access to cancer treatment for all ICERs, and no other published CEA made this assumption. Consequently, the between study variation for this variable competed with the random effects. In the logistic regression estimates without this variable, the coefficient estimates for the other covariates did not change. Sensitivity decreased from 0.390 to 0.338, but the model performance did not otherwise improve. The predicted probabilities did not increase substantially.

To further evaluate model fit, we grouped the country-level data from the Tufts registries into GBD super-regions, and calculated the proportion of ratios within a super region that were cost saving ratios. We then compared these values with the mean predicted probabilities from the logistic regressions model (**Table S4.3**). The proportion of ratios that were cost saving is lower in column 3 than column 2, because that subsample excludes the six studies that assumed 100% access to cancer treatment. Column 4 presents the mean probabilities for the Tufts sample using the mean value for all covariates. Results in column 4 are similar to column 2, although the probabilities in column 4 are smaller than the proportions in column 2 for Sub-Saharan Africa, High Income, and Latin American and the Caribbean super-regions, and larger than the proportions for others. The probabilities in column 5 are substantially smaller, because the predictions don’t use the mean value of the covariates. In particular, vaccine cost in the predictions was higher than the mean values in the published CEA (**S5 Appendix**), and the predictions assume payer perspective rather than social perspective. The predictions also assume that the HPV vaccine is given to females only, which would increase the probability that the result would be cost-saving.

| **Table S4.3. Summary of cost-saving ICERs by super-region in Tufts data and logistic regression out-of-sample predictions** | | | | |
| --- | --- | --- | --- | --- |
| **Super Region**  **(1)** | **Proportion of cost-saving ratios from Tufts data**  **(2)** | **Proportion of cost-saving ratios from Tufts data (assuming <100% access to care)**  **(3)** | **Mean predicted probability from Tufts data**  **(4)** | **Mean predicted probability for 195 countries**  **(5)** |
| Sub-Saharan Africa | 0.116 | 0 | 0.098 | 0.001 |
| High Income | 0.082 | 0.077 | 0.004 | < 0.001 |
| Southeast Asia, East Asia, and Oceania | 0.039 | 0.037 | 0.052 | < 0.001 |
| Latin America and Caribbean | 0.073 | 0.128 | 0.030 | < 0.001 |
| Central Europe, Eastern Europe, and Central Asia | 0 | 0 | 0.041 | < 0.001 |
| North Africa and Middle East | 0.026 | 0 | 0.072 | < 0.001 |
| South Asia | 0.032 | 0 | 0.067 | < 0.001 |

Once the model was used to make country-level predictions for all 195 countries, we also compared the mean predicted probabilities from the country-level predictions with the Tufts data (**Table S4.4-S4.5**) with the predictions for the 195 countries **(Table S4.6).**

| **Table S4.4.** Summary of predicted cost-saving probabilities for Tufts data, including random effect. | | | | | |
| --- | --- | --- | --- | --- | --- |
| Minimum | 25^th^ percentile | Median | Mean | 75^th^ percentile | Maximum |
| < 0.001 | < 0.001 | 0.001 | 0.074 | 0.022 | 0.994 |

| **Table S4.5.** Summary of predicted cost-saving probabilities for Tufts data, excluding random effect. | | | | | |
| --- | --- | --- | --- | --- | --- |
| Minimum | 25^th^ percentile | Median | Mean | 75^th^ percentile | Maximum |
| < 0.001 | < 0.001 | < 0.001 | 0.056 | 0.066 | 0.976 |

| **Table S4.6.** Summary of out-of-sample predicted cost-saving probabilities for 195 countries, random effect excluded. | | | | | |
| --- | --- | --- | --- | --- | --- |
| Minimum | 25^th^ percentile | Median | Mean | 75^th^ percentile | Maximum |
| < 0.001 | < 0.001 | < 0.001 | < 0.001 | < 0.001 | 0.004 |
