# Supplementary material for: Cost-effectiveness of HPV vaccination in 195 countries: A meta-regression analysis
Source: PLoS One. 2021 Dec 20;16(12):e0260808. doi: 10.1371/journal.pone.0260808 (PMC8687557; doi:10.1371/journal.pone.0260808)
Supplement: S5 Appendix — (DOCX) [file pone.0260808.s007.docx]

**S5 Appendix: Vaccine cost for predictions**

Vaccine cost is a covariate in the metaregression model, and we need a variable for vaccine cost for each country to predict its ICER. We used the HPV vaccine per dose as reported to the WHO’s Market Information for Access to Vaccines (MI4A)[1] and aggregated by Linksbridge.[2] Linksbridge reports five categories of prices per dose for the HPV vaccine in 2017. (**Table S5**). Vaccine cost was defined as the cost to completely vaccinate one person with a bivalent HPV vaccine in 2017. For example, the vaccine cost was US$13.50 ($4.50 x 3 doses) for countries that were eligible for GAVI support in 2020.

| **Table S5**. **Vaccine cost for estimating incremental cost-effectiveness ratios for 195 countries** | | |
| --- | --- | --- |
| **Category** | **Definition** | **2017 price per dose in US$** |
| United States | Price from the US Centers for Disease Control and Prevention | 137.72 |
| High income countries other than the United States | Countries classified as high income by the World Bank | 52.42 |
| Upper-middle income countries | Countries classified as upper-middle income by the World Bank that are not members of PAHO or eligible for UNICEF | 40.13 |
| Lower-middle income countries | Countries classified as lower-middle income by the World Bank that are not members of PAHO or eligible for UNICEF | Only 3 countries in this category were not eligible for PAHO and UNICEF, and we used the price for upper middle income countries |
| Pan American Health Organization (PAHO) | Countries eligible for the PAHO Revolving Fund for supported countries and vaccines | 9.15 |
| United Nations Children’s Fund (UNICEF) | UNICEF as price paid for purchase made for GAVI-supported countries and vaccines | 4.50 |

Linksbridge reports vaccine cost data without adjusting prices to the currency year of the most recent almanac. We use 2017 vaccine cost, which would be in 2017 US$.

We also explored fitting a regression model to the vaccine cost data reported in published CEA, but decided that the Linksbridge results are more representative. The vaccine cost in the published CEA are below US$10 per for about half of the published estimates, including many middle and high-income countries. **Figure S5** shows the relationship between vaccine cost and gross domestic product (GDP) per capita on a log scale for both sources of vaccine information: 1) published articles in the Tufts registries, and 2) Linksbridge. The low vaccine cost in the published articles is not a problem for the metaregression analyses, because the published ICERs are correspondingly low. They are a problem for predicting vaccine cost, because models don’t fit these data well, and vaccine cost is not representative of market prices.


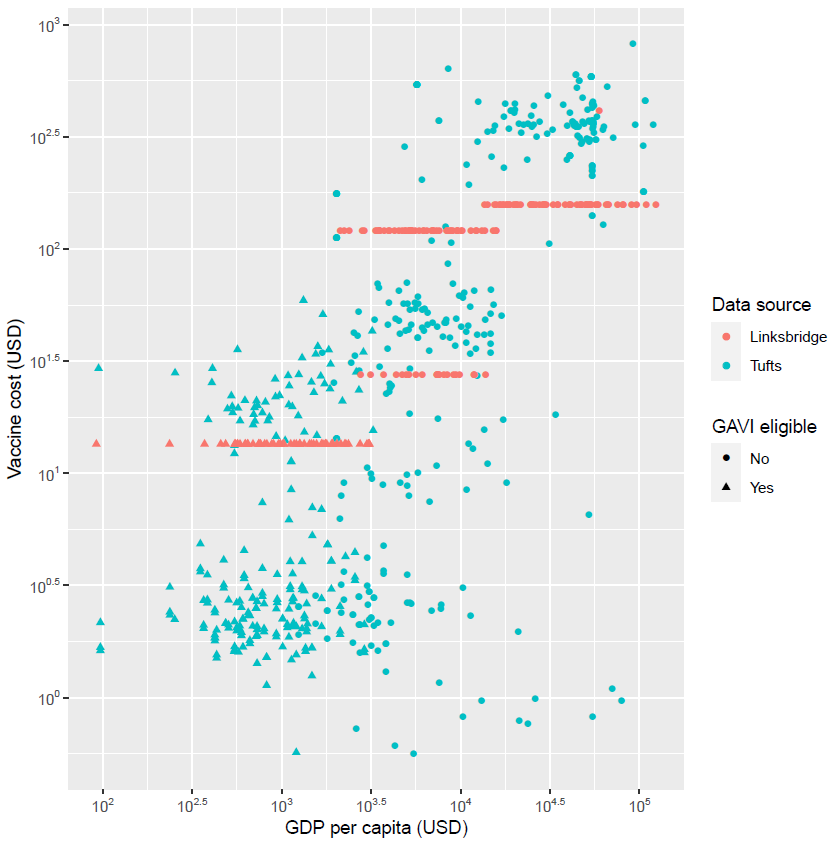


**Figure S5: Scatterplot of log vaccine cost versus log GDP per capita from sources of vaccine information: 1) published articles in the Tufts registries, and 2) Linksbridge**

A limitation of the Linksbridge results however, is the low response rate among high income countries on the MI4A reporting. For example in 2019, 41% of high income countries reported complete data, compared to 72% of countries globally.[3] The Mi4A data are more representative for other categories.

**Appendix S5 References**

1. World Health Organisation. MI4A vaccine purchase data for countries. 2018 Oct [cited 2021 Oct 24]. In: Market Information for Access to Vaccines (MI4A) [Internet]. Available from: https://www.who.int/publications-detail-redirect/mi4a-vaccine-purchase-data-for-countries

2. Linksbridge. Vaccine Almanac. 2021 Mar [cited 2021 Oct 24]. In: Global Vaccine Market Model (GVMM) [Internet]. For more information, please email gvmm@linksbridge.com

3. World Health Organization. Global vaccine market report. 2018 Oct [cited 2021 Oct 24]. In: Market Information for Access to Vaccines (MI4A) [Internet]. Available from: https://apps.who.int/iris/handle/10665/311278
