# Supplementary material for: Cost-effectiveness of HPV vaccination in 195 countries: A meta-regression analysis
Source: PLoS One. 2021 Dec 20;16(12):e0260808. doi: 10.1371/journal.pone.0260808 (PMC8687557; doi:10.1371/journal.pone.0260808)
Supplement: Attachment — Submitted filename: Response to reviewers.docx [file pone.0260808.s008.docx]

Response to reviewers

Editor comments on May 10, 2021

1.0 The minor revisions are in style, I consider their results interesting, so I suggest in the regression metadata analysis approach consider the following references that could help to strengthen your manuscript. a) Dios-Palomares, R. (2015). 7. Analysis of the Efficiency of Farming Systems in Latin America and the Caribbean Considering Environmental Issues. Revista Cientifica-Facultad de Ciencias Veterinarias, 25(1). b) Blanco-Orozco, N., Arce-Díaz, E., & Zúñiga-Gonzáles, C. (2015). Integral assessment (financial, economic, social, environmental and productivity) of using bagasse and fossil fuels in power generation in Nicaragua. Revista Tecnología en Marcha, 28(4), 94-107. c) Zuniga González, C. (2020). Total factor productivity growth in agriculture: Malmquist index analysis of 14 countries, 1979-2008. Revista Electrónica De Investigación En Ciencias Económicas, 8(16), 68-97. https://doi.org/10.5377/reice.v8i16.10661.

Response: Thank you for recommending these references. The data envelopment analyses reported in Dios-Palomares et al, and Zuniga-Gonzalez are an alternative to the stochastic frontier analysis (SFA) methods. While these aspects are outside of the scope of current work, where we do not use efficiency frontiers, we are very interested in these topics in the context of parallel projects in global health, where SFA and alternatives are used to quantify efficiency of interventions and funding of health initiatives. Together with co-authors Aravkin and Peng, we are working on a meta-analytic extension for SFA, where the references you sent are squarely in the scope of related methods. Thank you for these references, and we will be glad to keep you updated on SFA methods as we make progress.

Journal requirements on May 10, 2021

2.1. Please ensure that your manuscript meets PLOS ONE's style requirements, including those for file naming. The PLOS ONE style templates can be found at

https://journals.plos.org/plosone/s/file?id=wjVg/PLOSOne_formatting_sample_main_body.pdf and

<https://journals.plos.org/plosone/s/file?id=ba62/PLOSOne_formatting_sample_title_authors_affiliations.pdf>

Response: The title page and main body of the manuscript of the revised manuscript have been formatted to meet PLOS ONE’s style requirements, including file names.

2.2. Please review your reference list to ensure that it is complete and correct. If you have cited papers that have been retracted, please include the rationale for doing so in the manuscript text, or remove these references and replace them with relevant current references. Any changes to the reference list should be mentioned in the rebuttal letter that accompanies your revised manuscript. If you need to cite a retracted article, indicate the article’s retracted status in the References list and also include a citation and full reference for the retraction notice.

Response: To our knowledge, no references have been retracted, but some have been updated. All references in the main text have been formatted according to PLOS ONE’s style requirements. One published media reference was replaced with a citation to an available website.

2.3. We note that you have stated that you will provide repository information for your data at acceptance. Should your manuscript be accepted for publication, we will hold it until you provide the relevant accession numbers or DOIs necessary to access your data. If you wish to make changes to your Data Availability statement, please describe these changes in your cover letter and we will update your Data Availability statement to reflect the information you provide.

Response: We will provide accession numbers or DOI to access our data as stated.

2.4. We note that Figure 3 in your submission contain map images which may be copyrighted. All PLOS content is published under the Creative Commons Attribution License (CC BY 4.0), which means that the manuscript, images, and Supporting Information files will be freely available online, and any third party is permitted to access, download, copy, distribute, and use these materials in any way, even commercially, with proper attribution. For these reasons, we cannot publish previously copyrighted maps or satellite images created using proprietary data, such as Google software (Google Maps, Street View, and Earth). For more information, see our copyright guidelines: http://journals.plos.org/plosone/s/licenses-and-copyright.

We require you to either (1) present written permission from the copyright holder to publish these figures specifically under the CC BY 4.0 license, or (2) remove the figures from your submission:

Response: The maps in Figures 3A and 3B are original content that was created with open source software (R package ggplot2), and map boundaries from DAGM. According to the DAGM website, ““The data are freely available for academic use and other non-commercial use. Redistribution, or commercial use is not allowed without prior permission.” The following paragraph was added to the revised Modeling approaches subsection:

“The meta-regression analysis was performed with an open source mixed effects package <https://github.com/zhengp0/limetr> [20]. ICERs for 195 countries with adjustments were predicted with Python version 3.0 (Python Software Foundation, available at [http://www.python.org](http://www.python.org/)). The logistic regression analysis was performed using the open source software lme4 in R version 4.0.5 (Comprehensive R Archive Network, available at <https://cran.r-project.org/bin/windows/base/>). The maps in Figures 3A and 3B are original content that was created with open source software ggplot 2 in R package 4.0.5, and map boundaries from DAGM (Database of Global Administrative Areas, available at <https://gadm.org/data.html>).”

2.5. Please include captions for your Supporting Information files at the end of your manuscript, and update any in-text citations to match accordingly. Please see our Supporting Information guidelines for more information: http://journals.plos.org/plosone/s/supporting-information.

Reviewers' comments: A List of supporting information with files names and titles has been added to the revised manuscript. The in-text citations have been updated to match the file names.

Journal Requirements on October 27, 2021

2.6. We note that the grant information you provided in the ‘Funding Information’ and ‘Financial Disclosure’ sections do not match.

When you resubmit, please ensure that you provide the correct grant numbers for the awards you received for your study in the ‘Funding Information’ section.

Response: The two grant numbers in the Funding Information section are correct: OPP1152504, and OPP51229. The former grant number should be added to the Financial Disclosure section. May I ask you to please add this information? It is not part of the manuscript submission process, and so I can’t correct it.

2.7. Please upload a new copy of Figure 2 as the detail is not clear. Please follow the link for more information: <https://blogs.plos.org/plos/2019/06/looking-good-tips-for-creating-your-plos-figures-graphics/>

Response: We verified that Fig 2 adheres to the six criteria specified in the website, and uploaded it with the revised submission.

2.8. Please include a copy of Table S1 which you refer to in your text on page 5.

Response: S1 Table has been uploaded to the revised submission.

Reviewer #1:

3.1. The author must add a sub section in the methods parts which is about Meta Regression analysis. Otherwise, the metholodogical part of your paper become weak.

Response: In the revised subsection on Modelling approaches, we expanded the description of the five stages of the mixed-effects meta-regression framework from one paragraph to six, with a paragraph devoted to each of the five stages, as reproduced below:

“The statistical model and fitting procedures for the analysis of ICERs was conducted in five stages, and used a mixed-effects meta-regression framework (MR-BRT).^20^ This model included priors on all covariates and a study-specific random intercept. Each stage is described briefly below; for further information, see S3 Appendix.

“In the first stage, we estimated priors for selected covariates by leveraging the fact that one-way sensitivity analyses differ in no unmeasured covariates from their reference analyses. Four covariates had a sufficient number of sensitivity analyses reported published CEA to estimate priors using crosswalk models: vaccine cost, vaccine coverage, cost discount rate, and discount rate for health outcomes. We matched each sensitivity analysis with its corresponding reference analysis, and the crosswalk model estimated the difference in log-ICERs between sensitivity and reference analyses as a function of the difference between values of that covariate. We then constructed Gaussian priors for these covariates to use in all subsequent stages of the analysis with means and standard deviations equal to the crosswalk parameter estimates and standard errors from these crosswalk models.

“In the second stage, we estimated a nonlinear response curve for log-GDP per capita by modeling the log-ICERs as a nonlinear function of log-GDP per capita. Log cervical cancer DALYs per capita was entered linearly into this model, in addition to the four covariates addressed in the first stage, and the priors calculated in the first stage were placed on the corresponding covariates. To make this stage more robust to model misspecification, we placed a spline ensemble on log GDP per capita. This model also used a robust statistical approach for outlier detection, and outliers trimmed at this stage were discarded from subsequent steps of the analysis. The nonlinear response curve estimated by this model was used to transform log-GDP per capita for use in subsequent stages of the analysis.

“In the third stage, we selected additional covariates to include in the final meta-regression using a generalized Lasso approach for linear mixed effects models. The four crosswalk covariates, log cervical cancer DALYs per capita, and spline-transformed log-GDP per capita were pre-selected covariates at this stage, and the priors estimated for the crosswalk covariates were placed on those covariates. This process selected from nine additional candidate covariates: target sex, the proportion of model population assumed to have access to cervical cancer treatment, vaccine type, perspective, time horizon, comparator, and the outcome measure, and whether or not the intervention included a booster dose. Only one of these covariates, the assumption of a booster dose, was not selected for inclusion in the final model.

“In the fourth stage we selected the standard deviation of a Gaussian prior to apply to all regression parameters other than the intercept and the parameters for the four crosswalk covariates. To select a standard deviation, we fit a mixed effects meta-regression models with random intercepts by study, and priors on crosswalk covariates as calculated in the first stage. We normalized all other covariates and included Gaussian priors on those covariates, centered at zero and with a standard deviation that was constant across covariates. We varied this standard deviation using a grid-search and used 10-fold cross-validation to select the standard deviation that minimized the mean squared error for predicting values in the holdout set. We then converted the prior standard deviation back to the unstandardized scale for each covariate.

“In the fifth stage, we fit a mixed effects model with a random intercept and priors on covariates determined in the first and fourth stages. This model included priors on covariates calculated in the first and fourth stages and the transformed version of log-GDP per capita, and random intercepts by study.”

3.2 Also, the conclusion part must be improved

Response: The conclusion has been revised as follows:

This is the first attempt to generate a complete and consistent set of ICERs for HPV vaccines with UI for 195 countries. Meta-regression analysis can be conducted on CEA, where the one-way sensitivity analyses are used to quantify the effects of factors at the intervention and method-level. There is substantial uncertainty in the predicted ICERs in some countries, due to underlying heterogeneity of published CEA. Our results however, identified countries where the HPV vaccine is a good value, despite the uncertainty, and can facilitate decision-making across a wide range of settings.

Globally, introducing the HPV vaccine and achieving high HPV vaccine coverage are critical steps to eliminating cervical cancer burden. Building on all available information, our results support introducing and expanding HPV vaccination, especially in many countries that are eligible for subsidized vaccines from Gavi, the Vaccine Alliance, and the Pan American Health Organization. Vaccine cost is a key covariate, and our estimated models can be readily predictions ICERs and UI whenever vaccine subsidies are extended to additional countries or the vaccine price changes.

Reviewer #2: The manuscript seems interesting . The authors have conducted a thorough literature review, and analysed information accurately and sufficiently. However, the manuscript needs revisions.

4.1. Different formats have been adopted to quote references, the style should be according to the PLOS requirements and uniform.

Response: Thank you for this helpful feedback. The format for citing references complies with the PLOS One requirements in the revised manuscript.

4. 2. The policy implications need attention. There is an ample room to suggest more policy implications in the conclusion section.

Response: Please see the response to comment 3.2 above.
